# The Swiss franc safety premium

**DOI:** 10.1186/s41937-017-0014-7

**Published:** 2018-04-17

**Authors:** Jessica Leutert

**Affiliations:** Department of Economics, University of Lausanne, Lausanne, Switzerland

**Keywords:** Exchange rates, Safe haven currency, Swiss franc, F31, G12, G15

## Abstract

This paper applies a recent method proposed by Maggiori (*The U.S. Dollar Safety Premium*, 2013) to estimate the Swiss franc safety premium. The results show that the three-step instrumental variable approach as used by Maggiori does not work for the Swiss franc exchange rates. The price of risk estimates take unrealistic, negative values. One possible explanation is that the approach as it is used by Maggiori suffers from a measurement error for the expected exchange rate which represents a potential source of imprecision. By using the prediction of an augmented Fama regression to measure the expected exchange rate change, this measurement error can be avoided and the safety premium estimates become more realistic and closer to those obtained with a maximum likelihood-estimated GARCH approach. Overall, however, the GARCH approach still seems to be preferable to the instrumental variable approach.

**Electronic supplementary material:**

The online version of this article (10.1186/s41937-017-0014-7) contains supplementary material, which is available to authorized users.

## Introduction

The recent financial crisis and the subsequent European sovereign debt crisis provoked a large flight to quality among investors and caused strong upward pressure on the Swiss franc (CHF). It appreciated against the euro (EUR) by almost 40% within the relatively short time span of only 3 years. Major exchange rate interventions by the Swiss National Bank did not lead to the desired tension release, so that in September 2011, fearing an overvaluation of its currency, the Swiss central bank announced a lower bound of 1.20 on the EUR/CHF exchange rate.

A currency that has a general tendency to appreciate during episodes of intense crisis and offer hedging value against global risk is a currency that we would expect to earn a safety premium, defined as the compensation that investors require to short a safe currency and invest in a basket of foreign currencies. And as the willingness to short a safe currency decreases during risky episodes, we would expect this safety premium to be time-varying and to reach its highest values during periods of crises. One objective of this paper is to examine whether the Swiss franc earns a safety premium and to give an idea about its approximate size. Studying the potential safety premium of the Swiss franc might help to understand the dynamics of the Swiss franc exchange rate. Given Switzerland’s strong trade linkages with the rest of the world, variations in the Swiss franc exchange rate are not only an important factor in determining the profitability of Switzerland’s major export-oriented sector, but are also an important factor in determining domestic inflation. Hence, its dynamics will have major implications for monetary policy makers. Furthermore, the safety premium is a priced factor that can be reflected in many internationally traded assets.

While the focus of the recent empirical literature mainly lies on the analysis of unconditional safety premiums and ex-post currency excess returns, I make an attempt to calculate the time-varying Swiss franc safety premium, based on the conditional version of an International Capital Asset Pricing Model. An obvious way to estimate such a model would be to use a multivariate GARCH process, as has for example been done by De Santis and Gérard (1998). In a recent paper, however, Maggiori (2013) calculates the US dollar (USD) safety premium using another estimation methodology, based on the three-step instrumental variable approach developed by Duffee (2005). As compared to a GARCH approach, such a setup imposes less structure on the dynamics of the conditional covariance and has a higher flexibility. Maggiori (2013) presents promising results. In a first step, he calculates an estimate for the conditional covariance between a USD exchange rate index and the MSCI stock market return index. His results clearly show that this conditional covariance peaks in times of crisis, meaning that investors expect strong appreciations of the USD after negative stock market shocks. In a second step, Maggiori then estimates the risk price coefficient and finds a positive and significant value. By multiplying the conditional covariance by this risk price estimate, he gets an estimate for the USD safety premium, he finds the monthly USD safety premium to be around 10% in crisis times.

Applying Maggiori’s procedure to a trade-weighted Swiss franc exchange rate index and the EUR/CHF exchange rate, I expected to find similar patterns, but found results that are unsatisfactory. The conditional covariance estimates indeed confirm that investors expect the CHF to appreciate in times of crises, in other words, that they consider the CHF to be safe. At the same time, however, my results suggest that this safety is priced negatively, which is highly unrealistic. Given that investors are on average risk averse, theory and common sense tell us that the price of risk should be positive. Consequently, there seem to be some limitations in the methodology. So, a further objective of this paper is to provide a possible explanation and solution to these limitations. I argue that a potential problem lies in the construction of the dependent variable of the model: By definition, the safety premium is equal to the sum of the interest rate differential and the expected exchange rate change, which is unobservable. Maggiori suggests using the actual ex-post exchange rate change instead, which incorporates the prediction error made by investors. Given that forecasting exchange rates is difficult, this prediction error and hence the measurement error in the dependent variable are likely to be big. The solution to get around this problem is simple: The measurement error in the dependent variable can be avoided by choosing a different measure for the expected exchange rate change. I test two alternatives: The first one is to set the expected exchange rate change equal to zero, and the second one to set it equal to the prediction of an augmented Fama regression. For the Swiss franc, both options improve the results, but the second seems to dominate the first one. When using the prediction of the Fama regression, the risk price estimates become more realistic and closer to what I get when estimating the model, for comparison, with a multivariate GARCH specification, the Dynamic Conditional Correlation model by Engle (2002). Once a potential structural break in the relationship between Swiss franc exchange rate returns and equity returns in early 1999 is taken into account, these results reveal that the CHF safety premium is indeed time-varying, highest in times of crisis, and was equal to around 4.5% with peaks of up to 12.5% during the recent financial crisis, supporting the view of the CHF acting as a safe haven during periods of high risk.

Overall, my contribution shows that the three-step instrumental variable procedure proposed by Maggiori does not work for the Swiss franc and reveals a potential source of imprecision. I suggest a slight modification in the procedure that helps to improve the results for the Swiss franc. In my opinion, however, the instrumental variable approach still has some shortcomings compared to the maximum likelihood-estimated GARCH models: While maximum likelihood allows to estimate the model elegantly in one single step, the need for three separate steps to estimate it with instrumental variables is a source of impreciseness. Each individual step adds some uncertainty. Furthermore, an instrumental variable approach can only lead to convincing results when the available instruments are strong, which, at least in my sample, appears not to be the case.

The structure of the paper is as follows. The “Related literature” section gives an overview of the empirical literature on exchange rate returns and currency risk premiums. In the “Some descriptive evidence” section, I provide some descriptive evidence on the relationship between Swiss franc exchange rate changes and stock market returns. The “Safety premium model” section discusses the theoretical safety premium model. In the “Estimation strategy” section, I present the three-stage instrumental variable approach and in the “Data” section the data. The “Results” and “Time-varying price of risk” sections discuss the results and some extension. Finally, the “Comparison to a multivariate GARCH-in-mean specification” section compares the results to results obtained when using a GARCH specification and “Conclusions” section concludes and summarizes the main findings.

## Related literature

After the famous results by Fama (1984), the literature on risk premiums in the foreign exchange market experienced a first boom. The goal of testing for the presence of a time-varying currency risk premium was to deliver an alternative explanation for the failure of uncovered interest parity, as opposed to the explanation of simple failure of market efficiency. Prominent contributions were made by Hansen and Hodrick (1983), Hodrick and Srivastava (1984) and Domowitz and Hakkio (1985), to name only a few. All the three of them used the two-country model developed by Lucas (1982) as a theoretical foundation. Comprehensive surveys of the early literature on currency risk premiums are provided by Hodrick (1987) and Engel (1996). Overall, this evidence that departures from uncovered interest parity might be driven by risk premiums was rather mixed and received with considerable scepticism. The empirical contribution by Lustig and Verdelhan (2007) helped to increase the popularity of currency risk premium models again. Using a version of the consumption-based capital asset pricing model, they argue that risk associated with aggregate consumption growth can account for the differences in expected returns across different currency portfolios that are formed based on the size of the interest differential towards the US dollar. Burnside (2011), however, points out some critical features in Lustig and Verdelhan’s methodology and argues that one cannot reject the null hypothesis that their model explains none of the cross-sectional variation of the expected returns. Further examples of asset pricing models of currency returns with systematic deviations from UIP are estimated by Burnside et al. (2011), Lustig and Verdelhan (2011) and Lustig et al. (2014). The work by Maggiori (2013) and this paper differ from this recent literature insofar as the focus of the latter is mainly on the cross section of currency returns and unconditional moments. Maggiori, on the other hand, suggests a procedure to study the time-series properties of currency returns and allows to estimate the conditional currency safety premium.

An alternative approach for studying the properties of currencies and exchange rates is provided by the so-called factor models, where the sensitivity of ex-post currency returns to a set of risk factors is analysed. Burnside et al. (2006) find no significant covariance with a wide array of risk factors when analysing the returns to carry trade. Work using factor models to study the safe haven properties of currencies and in particular the Swiss franc appears to be more successful. Ranaldo and Söderlind (2010) estimate linear and non-linear factor models to study high-frequency exchange rates. Using risk factors that measure the performance of stock and bond markets as well as proxies for market volatility and liquidity, they find that the Swiss franc clearly exhibits the typical pattern of a safe haven currency as it tends to appreciate when there is an increase in risk. In addition, they document that there is some non-linearity in this pattern insofar as the appreciation of the Swiss franc is more than proportional to increases in risk and particularly strong during crisis episodes. Another study in this field is Hoffmann and Suter (2010), who examine the role of global and country-specific risk factors for exchange rates of the Swiss franc and find that it acts as a safe haven against some currencies, but not all. Grisse and Nitschka (2015) analyse the relationship between Swiss franc exchange rate returns and risk factors by estimating augmented UIP regressions. They find that the CHF exhibits safe haven characteristics against most other currencies. Furthermore, they also find significant time variation in the relationship between Swiss franc returns and the risk factors, with this link becoming stronger in times of stress. Finally, there is also empirical work analysing ex-post currency returns that has its main focus on the fact that an exchange rate’s comovement with falling markets might differ from its comovement with rising markets. Hossfeld and Macdonald (2015) for example document major differences in correlations between currency returns and global stock market returns conditional on the level of financial stress. Based on these observations, they explicitly distinguish between low and high stress regimes by estimating a threshold model. Controlling for the impact of carry trade reversal, they provide further evidence that the Swiss franc qualifies as a safe haven currency. A possible explanation for the relevance of this differentiation between rising and falling markets is provided by the literature on investors’ loss aversion (see for example Kahneman and Tversky (1979) and Gul (1991)). Ang et al. (2006) find evidence for the existence of a significant risk premium for holding stocks with high sensitivities to downside market movements and Atanasov and Nitschka (2014) find that downside risk is also priced in bilateral exchange rates.

## Some descriptive evidence

**Fig. 1:**
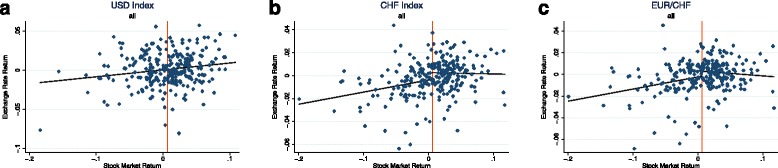
(**a**-**c**) Scatterplots—full sample. *Notes:* These scatterplots show the relationship between monthly domestic stock market returns and exchange rate returns (end of period values) for the time period January 1990 to August 2011. Domestic stock market returns are calculated from the S&P 500 Index in case of the USD exchange rate index and the SPI for the two CHF exchange rate

In their factor model analysis on exchange rates, Ranaldo and Söderlind (2010) define a safe haven currency to be a currency that offers hedging value against global risk, both on average and in particular so in crisis episodes. This implies that we generally should see a safe haven currency appreciate whenever stock market returns are low. In order to get a first idea on the relationship between the Swiss franc exchange rate and equity returns, and how it compares to the case of the US Dollar, Fig. 1 shows scatterplots of monthly currency returns versus domestic stock market returns (S&P 500 for the USD exchange rate index, SPI for the two CHF exchange rates) over the time period of January 1990 to August 2011^1^. These scatterplots reveal some interesting patterns. For the USD index, the relationship between currency returns and stock market returns seems to be fairly linear, while for the Swiss franc exchange rates, and particularly so for the EUR/CHF exchange rate, the relationship looks more hump-shaped: To low or negative stock market returns, the CHF appears to react by appreciating. On the other side, there seems to be no tendency of depreciations in the case of high stock market returns. One possible and common explanation for such a behaviour could be investors’ loss aversion. Important theoretical contributions in this field have been made by Kahneman and Tversky (1979) and Gul (1991) and suggest that investors care differently about downside losses than they care about upside gains. The guess about asymmetric behaviour in the CHF exchange rate is confirmed when estimating a very simple model:

1 $$  \triangle{e}_{t+1} = \alpha +\beta D_{t+1} + \gamma r^{\omega}_{t+1}+\delta D_{t+1} r^{\omega}_{t+1} + \epsilon_{t+1},  $$

where *e*_*t*_ corresponds to the (log) exchange rate defined as the price of the foreign currency in terms of home currency units and △*e*_*t*+1_ to the change in it. $r^{\omega }_{t+1}$ is equal to the stock market return, and  is a dummy equal to one when stock market returns are below the sample average and zero otherwise^2^. The coefficient *δ* thus measures the extent of asymmetry in the exchange rate reaction to high versus low equity returns. The model is estimated by OLS, using Newey-West standard errors in order to account for serial correlation. A Wald test is then performed to test the hypothesis of *β* and *δ* being jointly equal to zero. While this hypothesis is not rejected for the USD index, it is rejected at the 5% level for the CHF exchange rates, confirming the guess that the CHF reacts asymmetrically to high versus low stock market returns. The corresponding fitted lines are plotted in the CHF scatterplots. In the USD scatterplot, due to the insignificance of the asymmetry terms, the predicted line based on the model without dummy is plotted. The sample average of stock market returns is indicated by the red vertical line^3^.

**Fig. 2:**
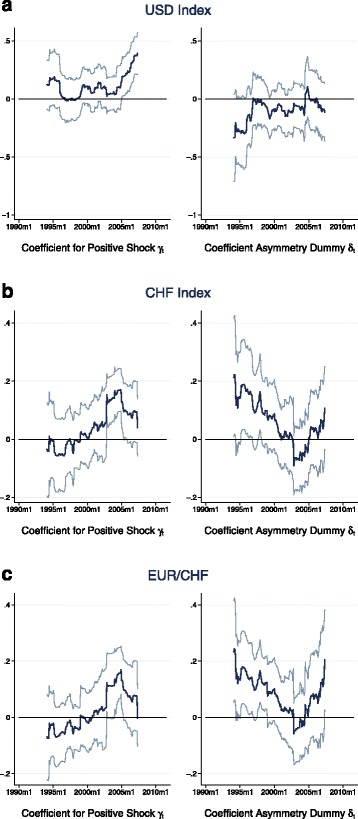
(**a**-**c**) Rolling estimation of asymmetry equation. *Notes:* These figures present the results of the rolling estimations of the equation $\triangle e_{t+1} = \alpha +\beta D + \gamma r^{\omega }_{t+1}+\delta D r^{\omega }_{t+1} + \epsilon _{t+1}$, where *D* is a dummy equal to one when stock returns are below their sample average and zero otherwise. The window width is set to 100. The model is estimated by OLS, using Newey-West standard errors with 3 lags. The resulting estimates for the *γ* and *δ* coefficients are plotted in dark color at the mid-point of each window. The light lines represent the 90% confidence intervals

Changes in the global and local economic and monetary environment can alter the role a currency plays in international financial markets. A major change in international monetary conditions was provoked by the introduction of the Euro in January 1999. This date thus appears to be an obvious candidate for a potential break. There is a large literature documenting this event and its consequences for other currencies. Fischer (2002) for example documents very moderate short-run volatility of the EUR/CHF exchange rate after the Euro introduction compared to previous episodes when the German mark was used as a benchmark. van Dijk et al. (2011) find evidence for large structural breaks in the unconditional correlations among the US dollar exchange rates of several currencies including the Swiss franc and the Euro. In order to test the stability of the relationship between the exchange rates and stock market returns in my sample, I perform a rolling estimation of Eq. (1) using a window width of 100 observations. At the mid-point of each window, Fig. 2 plots the rolling estimates of the correspondent *γ* and *δ* coefficients together with the according 90% confidence interval. The results raise indeed some doubts about the stability especially of the relationship between Swiss franc exchange rates and stock market returns. In early episodes, there seems to be no significant correlation between high stock market returns and CHF currency returns. On the other hand, low or negative stock market returns appear to go hand in hand with appreciations of the CHF, imposing an asymmetric behaviour of the Swiss franc exchange rate. In later periods, high stock market returns seem to be significantly accompanied by CHF depreciations, and with the *δ* parameter now being insignificant, low stock market returns will be accompanied by according (symmetric) CHF appreciations. In the case of the USD index, the *δ* parameter never gets significant, implying that there is no asymmetry present.

A Chow (1960) test performed on Eq. (1) to detect a potential structural break at the introduction of the euro in January 1999 finds supportive evidence at the 5% level for the EUR/CHF exchange rate^4^. Altogether, this break test and the results of the rolling estimation suggest that for further analyses incorporating the Swiss franc exchange rate, it might be necessary to split my sample into two subperiods^5^.

Finally, Table 1 shows the correlation between monthly exchange rate returns and equity returns for the different exchange rates and periods depending on whether the equity returns are below or above average. For the USD exchange rate index, there is no big difference between the two situations. The correlation is positive and significant both times, implying that the USD appreciates with stock market returns that lie below the average, and depreciates with returns above the average. For the trade-weighted Swiss franc exchange rate index and the EUR/CHF exchange rate, on the other hand, over the full sample and the first subsample, the correlation is highly and significantly positive in the case of low or negative equity returns, but much lower and insignificant in the case of high positive equity returns. In other words, these currency pairs see a significant appreciation of the Swiss franc when negative shocks hit the stock market, but no reaction of the exchange rate to positive shocks to the stock market^6^.

**Table 1:** Correlation table

	$Corr(r_{t+1}^{\omega }, \triangle e_{t+1})$	$Corr(r_{t+1}^{\omega }, \triangle e_{t+1})$	$Corr(r_{t+1}^{\omega }, \triangle e_{t+1})$
		for $r_{t+1}^{\omega } < \bar {r}$	for $r_{t+1}^{\omega } \geq \bar {r}$
USD Index			
*all*	0.18***	0.16*	0.23***
CHF Index			
*all*	0.32***	0.23**	−0.02
*T*<1999	0.25**	0.44***	0.03
*T*≥1999	0.37***	0.13	0.15
EUR/CHF			
*all*	0.27***	0.24**	−0.09
*T*<1999	0.13	0.46***	−0.03
*T*≥1999	0.38***	0.17	0.12

*Notes:* The first column of this table shows the unconditional correlation between monthly local stock market returns and exchange rate returns (end of period values) for the time period January 1990 to August 2011. The second and the third columns show the same correlation depending on whether stock returns are below or above their sample average. Local stock market returns are calculated from the S&P 500 Index in case of the USD exchange rate index and the SPI for the two CHF exchange rates

***, **, * denote significance levels of 1, 5, and 10%, respectively, based on a *t*-test

To summarize, the descriptive evidence provided in this section confirms that the CHF has clear safe haven tendencies vis-à-vis a trade-weighted basket of currencies and the euro in particular given the positive correlation of the exchange rate returns with the stock market returns. Hence, if investors care about risk, we would expect CHF exchange rates to, at least on average, incorporate a safety premium. However, there is also evidence that the relationship between stock market returns and exchange rate returns changed over time, namely, that it was subject to a structural break. This, in turn, suggests that it might be necessary to split my sample into two subperiods.

## Safety premium model

The theoretical model underlying the empirical estimations by Maggiori (2013) is based upon standard asset pricing theory in a complete market environment and starts with a simple no-arbitrage condition coming from an investor’s first-order conditions:

2 $$  {0}=E_{t} \left[ M_{t+1} \left(R_{t+1}^{*}\frac{\mathcal{E}_{t+1}}{\mathcal{E}_{t}} - R_{t+1} \right) \right].  $$

*M*_*t*+1_ denotes the home stochastic discount factor (SDF), *R*_*t*+1_ any asset return in the home country and $R_{t+1}^{*}$ a corresponding return in the foreign country (rest of the world). $\mathcal {E}_{t}$ is the exchange rate which is defined as the price of the foreign currency in terms of home currency units. An increase in $\mathcal {E}_{t}$ therefore corresponds to a depreciation of the home currency. According to Eq. (2), a home investor should expect a zero discounted excess return of investing abroad by shorting a home asset.

Assuming that asset returns, the stochastic discount factor and the exchange rate are jointly log-normally distributed, Eq. (2) can be linearized. Focusing on the case of risk-free interest rates, *R*_*f,t*+1_ and $R^{*}_{f,t+1}$, this yields (for the derivation see section A in the Additional file 1):

3 $$  \begin{aligned} {csp}_{t} &\equiv r_{f,t+1}^{*} + E_{t} \left[\triangle e_{t+1} \right] - r_{f,t+1} + \frac{1}{2} {Var}_{t} \left(\triangle e_{t+1} \right)\\ &= - {Cov}_{t} \left(m_{t+1}, \triangle e_{t+1} \right). \end{aligned}  $$

The lower case letters denote natural logarithms. The left-hand side of Eq. (3) is the expected excess log return of investing in the foreign risk-free asset by shorting the home risk-free asset plus Jensen’s inequality term. It defines the log currency safety premium *csp*_*t*_ that holders of the home currency have to pay. A currency is judged to be safe if it appreciates in times of economic distress and this is exactly what the right-hand side of Eq. (3) tells us: Times of economic distress are characterized by high marginal utility growth and thus a high stochastic discount factor. *csp*_*t*_ is positive if ${Cov}_{t} \left (m_{t+1}, \triangle e_{t+1} \right)$ is negative, thus if the home currency appreciates when the stochastic discount factor increases.

The most basic factor pricing model, the CAPM, is used to proxy for the stochastic discount factor: $m_{t+1} = a_{t} - b_{t} r_{t+1}^{\omega }$, where $r_{t+1}^{\omega }$ is the return on the investors’ benchmark portfolio, which is typically a market portfolio. Applying this substitution to Eq. (3), the currency safety premium will be positive if the currency’s appreciation is the higher the lower the market return:

4 $$  {csp}_{t} = - {Cov}_{t} \left(m_{t+1}, \triangle e_{t+1} \right) = b_{t} {Cov}_{t} \left(r_{t+1}^{\omega}, \triangle e_{t+1} \right).  $$

The size of the currency safety premium at a certain point in time *t* is determined by two components: The conditional covariance between the market return and the exchange rate change measures the time-varying quantity of risk an investor faces when investing into the foreign risk-free asset. The coefficient *b*_*t*_ tells us how much investors care about this risk and can thus be interpreted as the price of risk. It corresponds to the size of the safety premium in a case where the conditional covariance is equal to one. Theoretically, this price of risk can vary through time, but Maggiori takes it to be constant (*b*_*t*_=*b*), an assumption which I adopt. It will later be relaxed (see “Time-varying price of risk” section).

## Estimation strategy

### Three-stage instrumental variable approach

To estimate a currency’s safety premium according to Eq. (4), Maggiori (2013) suggests a procedure that relies on the three-stage instrumental variable approach developed by Duffee (2005) and is closely related to the instrumental variable approach of Campbell (1987) and Harvey (1989). The advantage of Duffee’s three-stage methodology is that it imposes only little structure on the dynamics of the conditional covariance and is therefore very flexible. The first two stages aim at estimating the conditional covariance ${Cov}_{t} \left (r_{t+1}^{\omega }, \triangle e_{t+1} \right)$. The goal of the third stage is to estimate the price of risk, *b*_*t*_.

To calculate the conditional covariance, one can use the fact that it can be expressed as the expected product of the prediction errors for the market return and the exchange rate change:

5 $$ {Cov}_{t} \left(r_{t+1}^{\omega}, \triangle e_{t+1} \right) = E_{t}\left[\eta_{t+1}^{r}\eta_{t+1}^{e}\right]  $$

where $\eta _{t+1}^{r}= r_{t+1}^{w} - E_{t}\left [ r_{t+1}^{w}\right ]$, and $\eta _{t+1}^{e}=\triangle e_{t+1} - E_{t}\left [ \triangle e_{t+1}\right ]$. In the zero-stage regressions, estimations for these prediction errors are calculated.^7^ Following an established literature based on Campbell and Shiller (1988), the time *t* expectation of the market return is modelled to linearly depend upon the log dividend-price ratio *dp*_*t*_. The time *t* expectation of the exchange rate change is modelled to linearly depend on the interest rate differential $\left (r_{f,t+1}^{*} - r_{f,t+1}\right)$ as suggested by Fama (1984). To account for possible serial correlation, one lag of the dependent variable is included in both cases:

6 $$\begin{array}{@{}rcl@{}} {} r_{t+1}^{\omega} &=& \alpha_{r0} + \alpha_{r1} {dp}_{t} + \alpha_{r2} r_{t}^{\omega} + \epsilon_{t+1}^{r}  \end{array} $$

7 $$\begin{array}{@{}rcl@{}} {} \triangle e_{t+1} &=& \alpha_{e0} + \alpha_{e1} \left(r_{f,t+1}^{*} - r_{f,t+1}\right) + \alpha_{e2} \triangle e_{t} + \epsilon_{t+1}^{e}  \end{array} $$

The product of the resulting residuals gives an ex-post estimate of the covariance: $ \widetilde {Cov_{t}} \left (r_{t+1}^{\omega }, \triangle e_{t+1} \right) \equiv \hat {\epsilon }_{t+1}^{r} \hat {\epsilon }_{t+1}^{e}$. It is important to notice that this object is based on time *t*+1 information. Hence, the goal of the next step is to make it conditional on time *t*. In the first-stage regression, the ex-post covariance estimate is projected on a set of time *t* instruments *Z*_*t*_:

8 $$\begin{array}{@{}rcl@{}} \widetilde{Cov_{t}} \left(r_{t+1}^{\omega}, \triangle e_{t+1} \right) &=& \alpha_{Z} Z_{t} + \xi_{t+1}  \end{array} $$

9 $$\begin{array}{@{}rcl@{}} \widehat{Cov_{t}} \left(r_{t+1}^{\omega}, \triangle e_{t+1} \right) &=& \hat{\alpha}_{Z} Z_{t}  \end{array} $$

The conditional covariance $\widehat {Cov_{t}} \left (r_{t+1}^{\omega }, \triangle e_{t+1} \right)$ is the estimate of the unobservable conditional covariance in Eq. (4). The set of possible instruments *Z*_*t*_ contains a constant, the dividend-price ratio, the lagged market return, the lagged exchange rate change, plus a measure for the lagged equity return variance, exchange rate return variance, and their covariance:

10 $$ Z_{t} = \left[1, {dp}_{t}, r_{t}^{\omega}, \triangle e_{t}, {var}_{t}^{{\prime}r}, {var}_{t}^{{\prime}e}, {cov}_{t}^{{\prime}} \right].  $$

According to Maggiori, these instruments have been found to reflect increases in risk premia in periods of stress. To calculate the lagged variances and covariance, Maggiori follows Duffee (2005) in calculating proxies that are independent of the zero-stage regressions:

11 $$\begin{array}{@{}rcl@{}} {var}_{t}^{{\prime}r}& \equiv& \sum\limits_{i=0}^{1} \left(r_{t-i}^{\omega}-\bar{r^{\omega}}\right)^{2} \; \; \; \; \; \; \; \; \; \; {var}_{t}^{{\prime}e} \equiv \sum\limits_{i=0}^{1} \left(\triangle e_{t-i}-\bar{\triangle e}\right)^{2} \end{array} $$

12 $$\begin{array}{@{}rcl@{}} {cov}_{t}^{\prime}& \equiv& \sum\limits_{i=0}^{2} \left(r_{t-i}^{\omega}-\bar{r^{\omega}}\right) \left(\triangle e_{t-i}-\bar{\triangle e}\right) \end{array} $$

where the barred values denote sample averages.

Finally, the second-stage regression aims at estimating the risk price coefficient *b*_*t*_ of Eq. (4) with instrumental variables. The price of risk is assumed to be constant through time:

13 $$  \begin{aligned} r_{f,t+1}^{*} &+ E_{t}\left[\triangle e_{t+1}\right] - r_{f,t+1} + \frac{1}{2} \widetilde{Var_{t}} \left(\triangle e_{t+1} \right)\\ &= b_{0} + b_{1} \widetilde{Cov_{t}} \left(r_{t+1}^{\omega}, \triangle e_{t+1} \right) + \omega_{t+1}. \end{aligned}  $$

$\widetilde {Var_{t}} \left (\triangle e_{t+1} \right) \equiv \left (\hat {\epsilon }_{t+1}^{e}\right)^{2} $ is the ex-post estimate of the variance of the exchange rate. In order to measure the unobservable conditional expectation of the exchange rate change $E_{t}\left [\triangle e_{t+1}\right ]$, Maggiori uses the actual ex-post exchange rate change △*e*_*t*+1_. In the next section, I will discuss this approach in more detail and make two alternative suggestions for how to proxy the expected exchange rate change.

The first-stage regression is estimated by OLS and corrects for possible heteroscedasticity and serial correlation by using Newey-West standard errors with the maximum lag order set equal to *T*^1/2^, i.e. the square root of the sample size (16 months for the full sample, 10 for the first and 12 for the second subsample). The zero-stage regression and second-stage regression are estimated jointly by GMM which allows the standard errors of the second-stage regression to incorporate not only the uncertainty deriving from the first-stage regression (as is common IV setups), but also the one from the zero-stage regression.^8^ Standard errors are based on the Newey-West estimate of the covariance matrix, with the maximum number of lags corresponding to the values of the first-stage regression.

### Alternative measures of the expected exchange rate change

In the empirical literature on risk and safety premiums, but also in the uncovered interest parity literature, there is a dependent variable that incorporates the expected exchange rate change, a variable that unfortunately is not observable. A common practice is to assume that the rational expectations hypothesis holds, so that forecast errors are uncorrelated with any time-*t* information. This allows to use the actual (ex-post) exchange rate change △*e*_*t*+1_ in place of the unobservable expected exchange rate change $E_{t} \left [ \triangle e_{t+1} \right ]$ to construct the dependent variable, with *e*_*t*+1_ being the future spot exchange rate. In cross-sectional studies where only the unconditional value of variables is of interest, the time-series average of the actual ex-post exchange rate return is likely to be a good approximation for its unconditional expected value. Namely, under the assumption that the exchange rate change is stationary and ergodic, a strong law of large numbers can be applied stating that the time-series average will almost surely converge to the unconditional expectation (see Karlin and Taylor (1975)): $T^{-1} \sum \triangle e_{t+1} \rightarrow E \left [ \triangle e_{t+1}\right ]$. In this case, there is no measurement error in the dependent variable. The situation is different, however, in time-series studies: Each observation consists of a single point in time, and using the actual ex-post exchange rate in place of the expected exchange rate implies that the dependent variable contains a measurement error consisting of the spread between the actual and the expected exchange rate change at a single point in time, the exchange rate prediction error $\eta _{t+1}^{e} = \triangle e_{t+1} - E_{t}\left [ \triangle e_{t+1}\right ]$. And this prediction error is likely to be big given that forecasting exchange rates is difficult. In the empirical literature, it has proven to be hard to find models that beat a simple random walk when considering short horizons. The prediction error and hence the measurement error that is incorporated in the dependent variable if the actual ex-post exchange rate is used to measure the expected exchange rate are therefore likely to be of similar magnitude as the actual exchange rate change itself.

What could be alternative and more appropriate measures for the expected exchange rate change in view of the actual exchange rate change generally being a bad proxy for the investors’ “real” exchange rate expectations and therefore potentially leading to imprecision? One option is to set the expected exchange rate change simply equal to zero (*E*_*t*_[△*e*_*t*+1_]=0), which would imply that investors believe that the exchange rate is following a random walk. Another option is to use the prediction of an augmented Fama model as it is estimated in the zero-stage regressions ($E_{t}\left [\triangle e_{t+1}\right ] = \widehat {E_{t}\left [\triangle e_{t+1}\right ]}$, see Eq. (7)). In the next sections, I am going to follow the estimation procedure suggested by Maggiori to estimate the conditional safety premium model, first using the actual exchange rate change as a proxy for the expected exchange rate change, and then using these two different measures.

## Data

Maggiori analyses monthly returns with his sample covering the period from January 1970 to March 2010. My analysis covers the much shorter period of January 1990 to August 2011, stopping at the time the Swiss National Bank introduced the lower bound for the EUR/CHF exchange rate^9^. This gives a total of 259 observations of which 107 are attributed to the first subsample covering the episodes before January 1999, while the remaining 152 observations are attributed to the second subsample. Maggiori builds exchange rate and interest rate differential indices for the US dollar using the MSCI World country weights incorporating 24 developed economies. These weights are constructed based on the market capitalization of the partner economies. This might be a reasonable weight when analysing the properties of the US dollar. When building these indices from the perspective of Switzerland, however, trade-flow shares might be an equally valid if not even better weight: Given Switzerland’s status as a heavily export-oriented economy, the policy-relevant exchange rate and thus the one of interest is rather the trade-weighted one. Moreover, the US dollar gets a weight of roughly 50% in the case of market capitalization-based weights and therefore definitely dominates the behaviour of such an index, which seems to be an undesirable property in the light of Switzerland’s strong linkages with the Euro area.

For this reason, I calculate trade-weighted indices for the Swiss franc. For the restricted period of 2006 to 2013, MSCI (Morgan Stanley Capital International) provided me with data on their MSCI World Index country weights comprising 24 developed economies which I all include in my index for the US dollar. By averaging over this sample, I create time-invariant weights^10^. Trade data for the construction of trade weights for the Swiss franc is taken from UN Comtrade. I create time-invariant trade weights for Switzerland by averaging over the whole sample period, equally weighting imports and exports. Only the five biggest trading partners are included in the index: The Euro area, the USA, the UK, China including Hong Kong, and Japan. Taken together, they make up for almost 80% of Switzerland’s trade volume. Euro area data which obviously is only available since the introduction of the Euro 1999 is merged with trade-weighted indices of exchange rates and interest rates of a sample of selected future Euro area countries to complete the series. A list of all countries considered in the construction of the indices can be found in the Appendix.

All data collected are end of period values. Bilateral spot exchange rates for building the indices are taken from IMF’s International Financial Statistics (IFS). For the risk-free interest rates, I use 1-month interbank rates from Datastream. I received early data on the STIBOR (Stockholm Interbank Offered Rate) from the Swedish National Bank directly. The S&P 500 Total Return Index, available on Datastream, and the Swiss Performance Index (SPI) Total Return Index, obtained from SIX Swiss Exchange, are taken to measure the benchmark market return of a US and Swiss investor, respectively. I chose to work with local stock market indices instead of global stock market indices in order avoid direct exchange rate effects that by construction appear in large international indices. Under the, given the financial openness of both the US and Switzerland not entirely unrealistic, assumption of complete markets, investors should anyway be indifferent about where to invest. Still, results based on the MSCI World index will be provided as a robustness test. The correlations between the local stock market indices and the MSCI World index (converted into the corresponding currency) are relatively high: 0.97 for the US and 0.83 for Switzerland. This suggests that the choice of the representative investor’s benchmark portfolio should in the end not make a big difference. Finally, I use the MSCI Dividend Yield Index to proxy for the dividend-price ratio (dividend yield is synonym for dividend-price ratio) of both these stock market indices. I obtained the corresponding data from MSCI directly.

Table 2 provides some descriptive statistics on (ex-post) excess returns over the time period between between January 1990 and August 2011. The average monthly excess return (annualized) is 1.37% of shorting the USD and investing in a basket of foreign currencies and −0.64 (−0.42)% of shorting the CHF and investing in a basket of foreign currencies (the euro). These negative values for the Swiss franc clearly reflect the fact that the latest month considered in my analysis, August 2011, lies right in the middle of the European sovereign debt crisis. When looking at a shorter time span going only until July 2007, also the Swiss franc currency trades exhibit positive average excess returns, suggesting that the average Swiss franc safety premium is positive. The largest monthly losses occurred in March 1991 for the USD (−7.8%), during Gulf War I, and in October 2008 for the CHF (around −6.5%), in the aftermath of the Lehman Brother collapse. The positive difference between foreign currency and CHF interbank rates explains the Swiss franc’s popularity as a carry funding currency.

**Table 2:** Descriptive statistics

	USD Index	CHF Index	EUR/CHF
Mean ExR (ann.)	1.38%	−0.54%	−0.30%
Mean up to 2007m7	0.93%	1.10%	1.34%
StD ExR	7.44%	5.51%	5.38%
Mean △*e*_*t*+1_	1.16%	−2.23%	−2.10%
Mean $r^{*}_{f,t+1}-r_{f,t+1}$	0.22%	1.69%	1.80%
Max (mon.)	5.77%	4.57%	4.76%
Max date	2009m5	2008m11	2008m11
Min	−7.78%	−6.16%	−6.68%
Min date	1991m3	2008m12	2008m10

*Notes:* Statistics are for monthly currency excess returns (ExR, defined as $\triangle e_{t+1} + r^{*}_{f,t+1}-r_{f,t+1}$) as well as for the corresponding subcomponents: Mean △*e*_*t*+1_ and mean $r^{*}_{f,t+1}-r_{f,t+1}$ are the average log exchange rate change and interest rate differential for each index. The total sample covers the period January 1990 to August 2011 (259 observations). The means and standard deviation are annualized, while the highest (Max) and lowest (Min) currency excess return realizations are on a monthly basis. The max and min date refer to the month when this highest and lowest returns occurred, respectively

## Results

This section presents the results I find when applying the GMM methodology to the USD and CHF exchange rate indices and to the EUR/CHF exchange rate.

### Zero-stage regression results

**Fig. 3:**
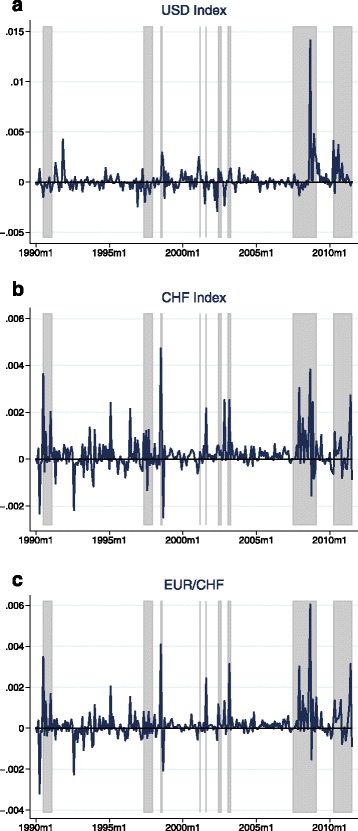
(**a**-**c**) Ex-post covariance—full sample. *Notes:* The estimates of the ex-post covariance are calculated as the product of the residuals of the zero stage regressions: $ \widetilde {Cov_{t}} \left (r_{t+1}^{\omega }, \triangle e_{t+1} \right) \equiv \hat {\epsilon }_{t+1}^{r} \hat {\epsilon }_{t+1}^{e}$, where $\hat {\epsilon }_{t+1}^{r}$ and $\hat {\epsilon }_{t+1}^{e}$ are the residuals in Eqs. (6) and (7). The zero stage regressions are estimated using the full sample, which consists of 259 observations

**Fig. 4:**
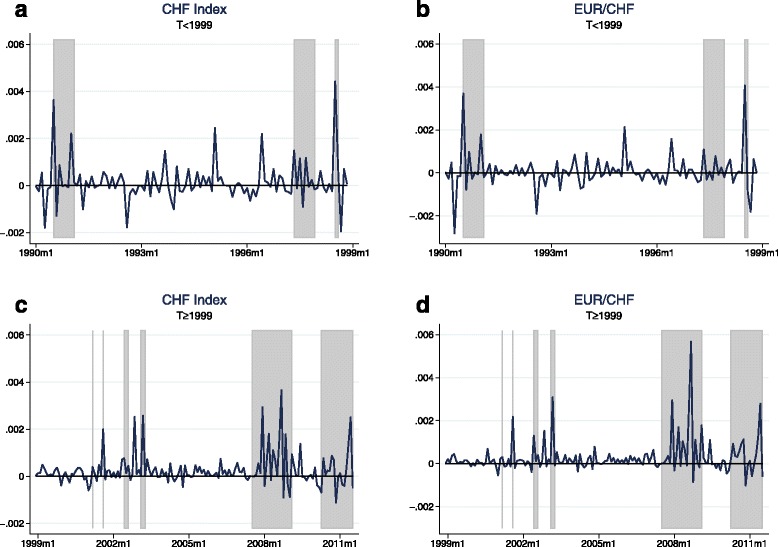
(**a**-**d**) Ex-post covariance—subsamples. *Notes:* The estimates of the ex-post covariance are calculated as the product of the residuals of the zero stage regressions: $ \widetilde {Cov_{t}} \left (r_{t+1}^{\omega }, \triangle e_{t+1} \right) \equiv \hat {\epsilon }_{t+1}^{r} \hat {\epsilon }_{t+1}^{e}$, where $\hat {\epsilon }_{t+1}^{r}$ and $\hat {\epsilon }_{t+1}^{e}$ are the residuals in equations (6) and (7). The zero stage regressions are estimated for each subsample separately. The first subsample (January 1990 to December 1998) consists of 107 observations and the second subsample (January 1999 to August 2011) consists of 152 observations

Tables 3 and 4 provide the results for the zero stage regressions. Unsurprisingly, given the short time horizon of only 1 month, the coefficients on the dividend price ratio in the equity return models are not statistically significant. However, they mostly have the expected sign and the value of the coefficient in the S&P 500 regression is close to Maggiori’s estimate in his MSCI regression^11^. The results on the exchange rate models are in line with the literature and confirm the common finding of the failure of UIP: Uncovered interest parity predicts a coefficient of −1 on the interest differential, while in the data one usually finds insignificant or even positive values^12^. In the case of the Swiss franc exchange rate indexes, my results even suggest highly and significantly positive values—a finding that may at least partly explain the Swiss franc’s role as a popular carry trade funding currency. The ex-post covariance is now calculated by multiplying the residuals of the equity returns regression by the residuals of the according exchange rate returns regression. Figures 3 and 4 plot the resulting estimates of the ex-post covariance for the full sample as well as the subsamples in case of the CHF exchange rates. The regions shaded in gray denote episodes of crisis manifested through high stock market volatility. A list of these crisis events is provided in Table 11 in the Appendix. The figures confirm that the USD appreciates during bad times in global stock markets. The same holds for the Swiss franc: There are positive spikes in the ex-post covariance at crisis episodes for both Swiss franc exchange rates, meaning that stock market slumps are accompanied by strong appreciations of the Swiss franc vis-à-vis the trade-weighted basket of currencies and, in particular, the euro.

**Table 3:** Zero-stage regression: equity returns

	S&P 500	SPI		
$r_{t+1}^{\omega }$	*All*	*All*	*T*<1999	*T*≥1999
*dp*_*t*_	0.012	0.005	−0.006	0.002
	[0.016]	[0.012]	[0.024]	[0.015]
$r_{t}^{\omega }$	0.089	0.217***	0.134	0.267***
	[0.089]	[0.044]	[0.084]	[0.054]
*Cons*	0.053	0.024	−0.010	0.007
	[0.061]	[0.047]	[0.087]	[0.059]
*R* ^2^	0.011	0.046	0.020	0.070

*Notes:* This table reports the results of regressing the equity return on the lagged log dividend-price ratio and the lagged equity return (see Eq. (6)). The parameters are estimated by OLS using Newey-West standard errors with maximum lag order set equal to *T*^1/2^. The number of observations is 259 for the full sample, 107 for the first subsample and 152 for the second subsample. The standard errors are reported in square brackets

***, **, * denote significance levels of 1, 5, and 10%, respectively

**Table 4:** Zero-stage regression: exchange rate returns

	USD Index	CHF Index			EUR/CHF		
△*e*_*t*+1_	*All*	*All*	*T*<1999	*T*≥1999	*All*	*T*<1999	*T*≥1999
$r^{*}_{f,t+1}-r_{f,t+1}$	0.147	5.447***	5.118***	7.750***	2.910*	2.419*	6.102*
	[0.820]	[1.326]	[1.848]	[2.818]	[1.507]	[1.365]	[3.503]
△*e*_*t*_	0.137***	−0.113	0.060	−0.249**	−0.110*	0.008	−0.187***
	[0.049]	[0.087]	[0.066]	[0.103]	[0.062]	[0.081]	[0.068]
*Cons*	0.001	−0.010***	−0.010**	−0.012***	−0.006***	−0.006*	−0.010**
	[0.001]	[0.002]	[0.004]	[0.004]	[0.002]	[0.003]	[0.004]
R^2^	0.019	0.054	0.062	0.089	0.028	0.018	0.055

*Notes:* This table reports the results of regressing the exchange rate return on the interest rate differential and the lagged equity return (see Eq. (7)). The parameters are estimated by OLS using Newey-West standard errors with maximum lag order set equal to *T*^1/2^. The number of observations is 259 for the full sample, 107 for the first subsample and 152 for the second subsample. The standard errors are reported in square brackets

***, **, * denote significance levels of 1, 5, and 10%, respectively

### First-stage regression results

Table 5 shows the results of the first-stage regressions, where the ex-post covariance is run on a set of time-*t* instruments in order to extract the predictable part. I exclude, unlike Maggiori, the lagged exchange rate change from my set of instruments *Z*_*t*_ in order to avoid an endogeneity problem once I use the zero stage prediction of the augmented Fama model to construct the dependent variable of the model. Instruments are required to be exogenous, meaning that they should effect the dependent variable only through their impact on the variable that is instrumentalized, a condition that would otherwise be violated.^13^ The covariance predictability is not very high, in case of the full sample estimations for the CHF exchange rates, the *R*^2^s are rather low and for the EUR/CHF exchange rate, the *χ*^2^-statistics is not rejected. The explanatory power improves and the predictability increases to significant levels when the CHF samples are split into the two subperiods, but the F-statistics which provide information about the relevance of instruments are still rather low. According to Staiger and Stock (1997), values that are far below 10 should raise some doubts concerning the strength of the instruments. By individually excluding some of the instruments, I manage to partially increase the F-statistic. For the sake of consistency across the different exchange rates and samples, however, I stick to the version including all the instruments. First-stage and second-stage results obtained when using such a limited set of instruments are provided in the Additional file 1: Section C.2. As it will turn out, the final results are almost equal and the relevant implications are unaffected by whether some possible instruments are excluded or not.

**Table 5:** First-stage regression

	USD Index	CHF Index			EUR/CHF		
$\widetilde {Cov_{t}} \left (r_{t+1}^{\omega }, \triangle e_{t+1} \right)$	*All*	*All*	*T*<1999	*T*≥1999	*All*	*T*<1999	*T*≥1999
*dp*_*t*_	0.001	0.000	−0.000	0.001**	0.000	−0.000	0.001**
	[0.000]	[0.000]	[0.001]	[0.000]	[0.000]	[0.001]	[0.000]
$r_{t}^{\omega }$	−0.004*	−0.000	0.001	−0.002	−0.001	0.001	−0.004*
	[0.002]	[0.001]	[0.001]	[0.001]	[0.001]	[0.001]	[0.002]
${var}_{t}^{{\prime }e}$	0.005	−0.046	−0.097	−0.177**	0.026	−0.092	−0.077
	[0.057]	[0.062]	[0.118]	[0.084]	[0.070]	[0.114]	[0.105]
${var}_{t}^{{\prime }r}$	0.016	−0.005	0.003	−0.016	−0.011	−0.006	−0.016
	[0.031]	[0.009]	[0.011]	[0.015]	[0.009]	[0.013]	[0.018]
${cov}_{t}^{\prime }$	0.061*	−0.071	−0.166**	0.034	−0.054	−0.133*	−0.025
	[0.033]	[0.053]	[0.068]	[0.061]	[0.057]	[0.080]	[0.074]
*Cons*	0.003	0.001	−0.001	0.003**	0.001	−0.001	0.004**
	[0.002]	[0.001]	[0.002]	[0.001]	[0.001]	[0.002]	[0.002]
*R* ^2^	0.088	0.021	0.092	0.082	0.023	0.093	0.104
F-statistic	12.08	2.231	4.285	2.447	1.660	7.478	2.947
*χ*^2^-statistic	60.400	11.155	21.425	12.235	8.300	37.390	14.735
*p* value (*χ*^2^-stat.)	0.000	0.048	0.001	0.032	0.140	0.000	0.012

*Notes:* This table reports the results of the first stage regression, which regresses the ex-post covariance obtained from the zero stage regressions on a set of instruments (see Eq. (8)). This set of instruments *Z*_*t*_ consists of a constant, the dividend-price ratio, the lagged equity return, plus a measure for the lagged equity return variance, exchange rate return variance, and their covariance. The parameters are estimated by OLS using Newey-West standard errors with maximum lag order set equal to *T*^1/2^. The F-statistic and the Wald *χ*^2^ test (plus the *p* value for the Wald *χ*^2^ test) are reported for the null hypothesis that all coefficients, except the constant, are jointly zero. The number of observations is 259 for the full sample, 107 for the first subsample and 152 for the second subsample. The standard errors are reported in square brackets

***, **, * denote significance levels of 1, 5, and 10%, respectively

**Fig. 5:**
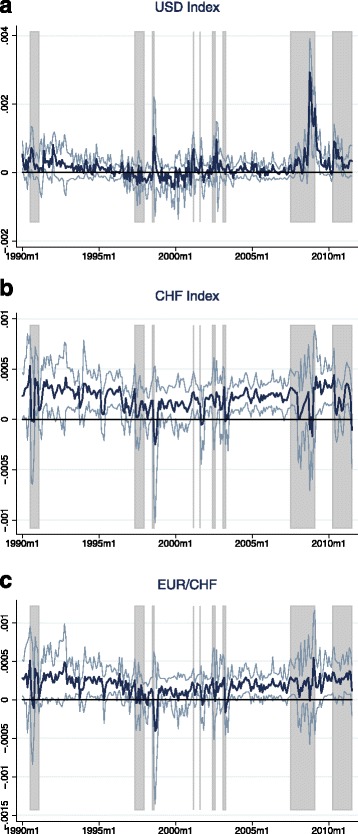
(**a**-**c**) Predicted conditional covariance—full sample. *Notes:* The estimates of the conditional covariance correspond to the fitted value of the first stage regression: $ \widehat {Cov_{t}} \left (r_{t+1}^{\omega }, \triangle e_{t+1} \right) = \hat {\alpha }_{Z} Z_{t}$ (see Eq. (9)). In this first stage regression, the ex-post covariance obtained from the zero stage regressions is regressed on a set of instruments. The set of instruments *Z*_*t*_ consists of a constant, the dividend-price ratio, the lagged equity return, plus a measure for the lagged equity return variance, exchange rate return variance, and their covariance. The first-stage regression is estimated using the full sample, which consists of 259 observations. The two thin lines represent the 95% confidence band and are based on a two sided t-statistic with Newey-West estimates of the standard errors

**Fig. 6:**
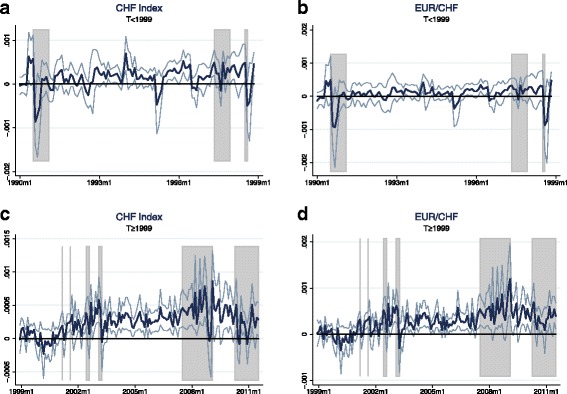
(**a**-**d**) Predicted conditional covariance—subsamples. *Notes:* The estimates of the conditional covariance correspond to the fitted value of the first stage regression: $ \widehat {Cov_{t}} \left (r_{t+1}^{\omega }, \triangle e_{t+1} \right) = \hat {\alpha }_{Z} Z_{t}$ (see Eq. (9)). In this first stage regression, the ex-post covariance obtained from the zero stage regressions is regressed on a set of instruments. The set of instruments *Z*_*t*_ consists of a constant, the dividend-price ratio, the lagged equity return, plus a measure for the lagged equity return variance, exchange rate return variance, and their covariance. The zero stage regressions are estimated for each subsample separately. The first subsample (January 1990 to December 1998) consists of 107 observations and the second subsample (January 1999 to August 2011) consists of 152 observations. The two thin lines represent the 95% confidence band and are based on a two sided t-statistic with Newey-West estimates of the standard errors

Figures 5 and 6 show the conditional covariance obtained from the predictions of these first-stage regression results along with the 95% confidence band. Even tough I am analysing a much shorter time span than Maggiori, my conditional covariance estimate for the USD exchange rate index is fairly close to his. The conditional covariance is clearly time-varying and spikes in times of crises as it can be seen for example in the case of the recent financial crisis, starting with the credit crunch in August 2007 and reaching a bottom with the default of Lehman Brothers in October 2008. For the CHF exchange rates, the pattern is less clear, especially for the full sample, which is not surprising given the low predictive power of the model. Only the figure for the second subsample gives plausible results with the conditional covariance being time-varying, significantly positive most of the time, and highest during the recent financial crisis.

### Second-stage regression results

The final step now aims at finding the price of risk *b* by estimating Eq. (13) applying a two-step GMM approach.^14^ Table 6 presents the results for the full sample, Table 7 for the subsamples. The estimated price of risk $\hat {b}$ corresponds to the coefficient on the $\widetilde {Cov_{t}} \left (r_{t+1}^{\omega }, \triangle e_{t+1} \right)$ variable. The first group of estimates in each table is attained by following Maggiori’s suggestion and setting the expected exchange rate change in the dependent variable by the actual ex-post exchange rate change: *E*_*t*_[△*e*_*t*+1_]=*e*_*t*+1_. The second and third groups of estimates are attained by setting the expected exchange rate change equal to zero (*E*_*t*_[△*e*_*t*+1_]=0) and by using the predicted exchange rate change from the zero-stage regression ($E_{t}\left [\triangle e_{t+1}\right ] = \widehat {E_{t}[\triangle e_{t+1}]} $). For the USD, I get positive numbers across all cases. Maggiori (2013) finds highly significant values of between 3 and 16 for the price of risk, while I find a significantly positive value of 6.491 when following him and measuring the expected exchange rate change by the ex-post exchange rate change.^15^ The risk price estimates are lower when using one of the alternative measures. For the CHF, on the other side, the risk price coefficient is negative when estimated with the actual exchange rate change in the dependent variable, in case of the full sample even at statistically significant levels. This finding would imply that investors demand a (positive) risk premium for holding a currency that has a positive conditional covariance, in other words, that they expect to appreciate when equity returns are low. However, a currency with a positive conditional covariance is a currency that helps them to smooth their consumption level and therefore should be considered as safe and attractive. Theory and common sense tell us that investors will be willing to pay a safety premium for such a currency, and not demand a risk premium, and especially, that the price of risk should be positive. These results raise serious doubts about the general validity of the methodology as it is applied by Maggiori, i.e. with the expected exchange rate change proxied by the actual ex-post exchange rate change. The estimates for the price of risk for the Swiss franc exchange rates improve when the model is estimated with one of the alternative measures for the expected exchange rate change. The risk price coefficients become more realistic and the respective standard deviations decrease^16^. The results for the full sample are still not convincing, the coefficients are still negative, albeit much smaller in absolute terms. Again, this is not so surprising given the weak performance of the instruments predicting the conditional covariance. For the subsamples, then, the price of risk estimates become a lot more plausible: When estimated with the zero stage prediction, they are positive across the two samples and exchange rates, rather close to each other (they are all around 2 to 4) and significant. Hence, limiting the measurement error incorporated in the dependent variable seems to help improve the results for the Swiss franc. Based on these estimates, the safety premium for the Swiss franc reflected in the EUR/CHF exchange rate during the recent financial crisis would be around 1% to maximally 3.5% (on an annual basis)^17^, a value that seems to be rather low in the light of the dramatic appreciations it experienced at the time and that might be attributed to the poor performance of the instruments. When the model is estimated with the “optimal” set of instruments (the subset of instruments that maximizes the F-statistic in the first stage regression, see Additional file 1: Section C.2), the safety premium reflected in the EUR/CHF exchange rate during the recent financial crisis is found to be around 2.5% on average and around 5% at the peak^18^.

**Table 6:** Second-stage regression—full sample

$r^{*}_{f,t+1}+E_{t}\left [\triangle e_{t+1}\right ]-r_{f,t+1}$	$E_{t}\left [\triangle e_{t+1}\right ] = \triangle e_{t+1}$			$E_{t}\left [\triangle e_{t+1}\right ] = 0 $			$E_{t}\left [\triangle e_{t+1}\right ] = \widehat {E_{t}[\triangle e_{t+1}]} $		
$ + \frac {1}{2} \widetilde {Var_{t}} \left (\triangle e_{t+1} \right)$	USD Index	CHF Index	EUR/CHF	USD Index	CHF Index	EUR/CHF	USD Index	CHF Index	EUR/CHF
$\widetilde {Cov_{t}} \left (r_{t+1}^{\omega }, \triangle e_{t+1} \right)$	6.491**	−22.703***	−15.975***	1.047***	−1.582**	−0.963***	0.992	−10.663***	−2.053
	[3.572]	[8.740]	[6.740]	[0.375]	[0.692]	[0.406]	[1.115]	[4.343]	[1.633]
*Cons*	0.000	0.004***	0.003**	0.000	0.002***	0.002***	0.001***	0.002**	0.000
	[0.001]	[0.002]	[0.001]	[0.000]	[0.000]	[0.000]	[0.000]	[0.001]	[0.000]
J-statistic	4.097	0.825	1.390	4.369	0.894	1.880	5.439	1.141	5.269
*p* value	0.393	0.935	0.846	0.358	0.925	0.758	0.245	0.888	0.261

*Notes:* This table reports the results of the second-stage regression for the case of no time variation in the price of risk (see Eq. (13)). The dependent variables are the USD and CHF safety premium, respectively, defined as the expected excess return of investing in the foreign risk-free asset by shorting the home risk-free asset. The expected exchange rate change used to calculate this expected excess return is proxied first by the actual exchange rate change, then by zero, and finally by the fitted value of the zero stage regression. The regressors are a constant and the estimate of the conditional covariance between stock returns and exchange rate changes from the first stage regression. The set of instruments *Z*_*t*_ consists of a constant, the dividend-price ratio, the lagged equity return, plus a measure for the lagged equity return variance, exchange rate return variance, and their covariance. The second-stage regression is estimated jointly with the zero stage regression by GMM which allows the standard errors of the second-stage regression to incorporate not only the uncertainty deriving from the first-stage regression, but also the one from the zero stage regression. The standard errors are based on the Newey-West estimate of the covariance matrix with maximum lag order set equal to *T*^1/2^. The J-statistic (Hansen 1982) plus the according *p* value are reported for the null hypothesis that the model is well-specified and the moment conditions do hold. The number of observations is 259 for the full sample. The standard errors are reported in square brackets

***, **, * denote significance levels of 1, 5, and 10%, respectively

**Table 7:** Second-stage regression—subsamples

$r^{*}_{f,t+1}+E_{t}\left [\triangle e_{t+1}\right ]-r_{f,t+1}$	$E_{t}\left [\triangle e_{t+1}\right ] = \triangle e_{t+1}$		$E_{t}\left [\triangle e_{t+1}\right ] = 0 $		$E_{t}\left [\triangle e_{t+1}\right ] = \widehat {E_{t}\left [\triangle e_{t+1}\right ]} $	
$ + \frac {1}{2} \widetilde {Var_{t}} \left (\triangle e_{t+1} \right)$	CHF Index	EUR/CHF	CHF Index	EUR/CHF	CHF Index	EUR/CHF
Subperiod 1						
$\widetilde {Cov_{t}} \left (r_{t+1}^{\omega }, \triangle e_{t+1} \right)$	−5.972	−7.086*	0.637***	0.718**	4.260***	2.482**
	[4.902]	[4.538]	[0.234]	[0.365]	[1.462]	[1.230]
*Cons*	0.001	0.002*	0.002***	0.002***	0.001*	0.001***
	[0.002]	[0.001]	[0.000]	[0.000]	[0.001]	[0.000]
J-statistic	3.745	2.804	5.887	4.561	6.225	4.632
*p* value	0.442	0.591	0.208	0.335	0.183	0.327
Subperiod 2						
$\widetilde {Cov_{t}} \left (r_{t+1}^{\omega }, \triangle e_{t+1} \right)$	−0.101	−1.530	0.050	0.328**	2.610*	3.211***
	[3.789]	[4.463]	[0.198]	[0.150]	[1.881]	[0.926]
*Cons*	0.000	0.000	0.001***	0.001***	−0.001***	−0.001***
	[0.001]	[0.001]	[0.000]	[0.000]	[0.000]	[0.000]
J-statistic	3.761	4.808	3.394	4.417	5.148	5.536
*p* value	0.439	0.308	0.494	0.353	0.272	0.237

*Notes:* This table reports the results of the second-stage regression for the case of no time variation in the price of risk (see Eq. (13)). The dependent variables are the USD and CHF safety premium, respectively, defined as the expected excess return of investing in the foreign risk-free asset by shorting the home risk-free asset. The expected exchange rate change used to calculate this expected excess return is proxied first by the actual exchange rate change, then by zero, and finally by the fitted value of the zero stage regression. The regressors are a constant and the estimate of the conditional covariance between stock returns and exchange rate changes from the first stage regression. The set of instruments *Z*_*t*_ consists of a constant, the dividend-price ratio, the lagged equity return, plus a measure for the lagged equity return variance, exchange rate return variance, and their covariance. The second-stage regression is estimated jointly with the zero stage regression by GMM which allows the standard errors of the second-stage regression to incorporate not only the uncertainty deriving from the first-stage regression, but also the one from the zero stage regression. The standard errors are based on the Newey-West estimate of the covariance matrix with maximum lag order set equal to *T*^1/2^. The J-statistic (Hansen 1982) plus the according *p* value are reported for the null hypothesis that the model is well-specified and the moment conditions do hold. The model is estimated for each subsample separately. The first subsample (January 1990 to December 1998) consists of 107 observations and the second subsample (January 1999 to August 2011) consists of 152 observations. The standard errors are reported in square brackets

***, **, * denote significance levels of 1, 5, and 10%, respectively

Remarkably, the price of risk estimates hardly change when the lagged exchange rate change is used as an instrument but not as a regressor in the Fama regression, or when the model is estimated with the “optimal” set of instruments (see the corresponding results in Additional file 1: Section C.2). They appear also to be quite robust across the two subsamples and the different CHF exchange rates. Thus, even tough there might be some concerns about the strength of the instruments and also the J-statistic (Hansen 1982), indicating in some, but not all cases that there might be some issues with the model specification, the CHF price of risk estimates seem to be credible to some extent.

### Robustness

A range of five robustness tests is presented and discussed in detail in the Additional file 1: I performed the estimations using different sample periods (including one going back to 1975 and one excluding the Global Financial Crisis and the Great Recession), a different (global) benchmark return and a financially weighted instead of trade-weighted CHF exchange rate index. Overall, all robustness tests support the above finding that proxying the expected exchange rate change by the prediction of the zero stage regression yields at least as or even more realistic and reliable estimations of the price of risk as compared to measuring the expected exchange rate change by the actual ex-post exchange rate.

## Time-varying price of risk

This section relaxes the assumption that the price of risk is constant through time. From a theoretical point of view, a constant price of risk would be justified by power utility, while the more realistic recursive utility and habit formation models imply that the price of risk may vary through time. There is evidence which finds that when studying the dynamics of economic risk premiums, the time-variation in the price of risk is more important than changes in the quantity of risk (see for example Ferson and Harvey (1991))^19^. With their BEKK model, also De Santis and Gérard (1998) only find the price of risk to be significant once they allow it to vary through time. I closely follow them in the parametrization of the risk price coefficient and in the choice of instruments. The time-varying price of risk *b*_*t*_ is modelled using a linear function:

14 $$ b_{t} = \kappa Y_{t}, \quad Y_{t} = \left[ 1, {dp}_{t}, d.{ys}_{t}, d.r_{f,t}, baa\mathrm{\_}{aaa}_{t} \right],  $$

where *κ* is a 1 ×5 vector. *Y*_*t*_ corresponds to a set of instruments including a constant, the market index dividend price ratio *dp*_*t*_ and the change in the gap between long-term and short-term interest rates (yield spread) *ys*_*t*_ measured by the yield of 10-year government bond in excess of the 1-month interbank rate. Furthermore, it includes the change in the home risk-free interest rate *r*_*f,t*_ and the yield difference of Moody’s BAA-rated corporate bonds over Moody’s AAA-rated corporate bonds *baa_aaa*_*t*_ (taken from FRED), which is used as a measure for default risk. This way of parameterizing the risk price allows to easily check for the time-variation of the coefficient by setting all *κ*’s except the first one (the one related to the constant) equal to zero.

The results of this extended model when estimating it by the three-step GMM methodology are presented in Table 8. The equation that is estimated is the following:

15 $$ {} \begin{aligned} r_{f,t+1}^{*} + E_{t}\left[\triangle e_{t+1}\right] - r_{f,t+1} = b_{0} + \kappa Y_{t} \widetilde{Cov_{t}} \left(r_{t+1}^{\omega}, \triangle e_{t+1} \right) + \omega_{t+1}, \end{aligned}  $$

**Table 8:** Second-stage regression—time-varying price of risk

$r^{*}_{f,t+1}+\widehat {E_{t}\left [\triangle e_{t+1}\right ]}-r_{f,t+1}$	*All*			*T*<1999		*T*≥1999	
$ + \frac {1}{2} \widetilde {Var_{t}} \left (\triangle e_{t+1} \right)$	USD Index	CHF Index	EUR/CHF	CHF Index	EUR/CHF	CHF Index	EUR/CHF
$\widetilde {Cov_{t}} \left (r_{t+1}^{\omega }, \triangle e_{t+1} \right)$	238.052*	−69.980	−111.617	−82.002	−55.257	−434.596*	−33.416
	[155.262]	[103.549]	[110.291]	[112.052]	[108.330]	[290.049]	[88.693]
$\widetilde {Cov_{t}} \left (r_{t+1}^{\omega }, \triangle e_{t+1} \right)*{dp}_{t}$	58.169*	−12.174	−25.785	−23.436	−14.114	−123.582*	−8.220
	[37.989]	[23.571]	[24.536]	[26.110]	[23.680]	[79.216]	[23.181]
$\widetilde {Cov_{t}} \left (r_{t+1}^{\omega }, \triangle e_{t+1} \right)*d.{ys}_{t}$	−1245.245	−1533.296	−2908.650*	1235.988	3613.389**	−6344.461*	−832.865
	[1677.153]	[3471.034]	[2199.215]	[1388.052]	[1660.386]	[4052.069]	[905.723]
$\widetilde {Cov_{t}} \left (r_{t+1}^{\omega }, \triangle e_{t+1} \right)*d.r_{f,t}$	−1727.834*	−4939.896*	−2991.115	−2342.645	5548.407***	−4157.170	94.303
	[1283.600]	[3510.700]	[3678.825]	[2073.427]	[1957.128]	[3612.278]	[967.194]
$\widetilde {Cov_{t}} \left (r_{t+1}^{\omega }, \triangle e_{t+1} \right)* baa\mathrm {\_}{aaa}_{t}$	−1776.649*	866.314	698.838	−2345.300	784.905	−382.272	202.512
	[1188.008]	[2014.862]	[1490.346]	[2138.844]	[2459.814]	[1902.112]	[556.533]
*Cons*	0.001	0.002	−0.000	0.002***	0.002***	−0.008**	−0.001
	[0.001]	[0.002]	[0.001]	[0.001]	[0.001]	[0.004]	[0.001]
J-statistic	1.142	4.532	3.373	4.474	2.480	3.932	5.919
*p* value (J-stat.)	0.767	0.209	0.338	0.215	0.479	0.269	0.116
*χ*^2^-statistic	5.194	4.624	7.656	9.964	15.077	2.881	5.603
*p* value (*χ*^2^-stat.)	0.268	0.328	0.105	0.041	0.005	0.578	0.231

*Notes:* This table reports the results of the second-stage regression allowing for time variation in the price of risk. The dependent variables are the USD and CHF safety premium, respectively, defined as the expected excess return of investing in the foreign risk-free asset by shorting the home risk-free asset. The expected exchange rate change used to calculate this expected excess return is proxied by the fitted value of the zero stage regression. The regressors are a constant, the estimate of the conditional covariance between stock returns and exchange rate changes from the first stage regression, as well as four terms where the conditional covariance is interacted with the dividend price ratio, the yield spread between long- and short-term bonds, the change in the home risk free interest rate and the yield difference between BAA- and AAA-rated bonds. The set of instruments consists of a constant, the dividend-price ratio, the lagged equity return, plus a measure for the lagged equity return variance, exchange rate return variance, and their covariance as well as the yield spread, the change in the home risk free interest rate and the yield difference, which are included for technical reasons. The second-stage regression is estimated jointly with the zero stage regression by GMM which allows the standard errors of the second-stage regression to incorporate not only the uncertainty deriving from the first-stage regression, but also the one from the zero stage regression. The standard errors are based on the Newey-West estimate of the covariance matrix with maximum lag order set equal to *T*^1/2^. The J-statistic (Hansen 1982) plus the according *p* value are reported for the null hypothesis that the model is well-specified and the moment conditions do hold. The Wald *χ*^2^ test plus the according *p* value are reported for the null hypothesis that all four coefficients on the interaction terms are jointly equal to zero. The number of observations is 259 for the full sample, 107 for the first subsample and 152 for the second subsample. The standard errors are reported in square brackets

***, **, * denote significance levels of 1, 5, and 10%, respectively

where *E*_*t*_[△*e*_*t*+1_] is proxied by the prediction of the augmented Fama regression $\widehat {E_{t}[\triangle e_{t+1}]}$. Hardly any of the coefficients on the interaction terms is statistically significant and the Wald tests only provide evidence for them to be jointly significant in the first subperiod. Overall, this suggests that allowing the price of risk to change through time does not help to improve the model, in fact it even seems to be a deterioration compared to the constant price of risk model, as there all price of risk coefficients are significantly positive in the subperiods.

## Comparison to a multivariate GARCH-in-mean specification

A straightforward drawback of the three-step GMM estimation procedure is the large number of orthogonality conditions that must hold for the estimation to be valid. While offering a high degree of flexibility, this makes this method also somewhat unreliable and is probably the reason why GMM has been given so little attention so far in the estimation of currency safety premiums. In finance, multivariate generalized autoregressive conditional heteroscedasticity (GARCH) models are by now a well-established method for calculating the covariance matrix of a conditional model. Maximum likelihood estimation under the assumption that the covariance matrix or the variances and covariances follow an autoregressive process allows to estimate the model in one single step and there is no need for instruments for calculating the conditional covariance.

This section presents the main results I obtain when the above safety premium model is for comparison estimated with a DCC^20^ multivariate GARCH-in-mean specification. A detailed exposition of the MGARCH set-up, the technical details of the estimation strategy and the complete results are provided in Additional file 1: Section D.

The equation of interest in the whole set-up is the currency excess returns mean equation:

16 $$\begin{array}{*{20}l} xr^{c}_{t+1} &= \gamma^{c}_{0} + \gamma^{c}_{1} \sigma_{c,m,t+1} + u^{c}_{t+1}, \end{array} $$

where $xr^{c}_{t+1}$ corresponds to the excess return of investing abroad $\left (=r_{f,t+1}^{*} + \triangle e_{t+1} - r_{f,t+1}\right)$, *σ*_*c,m,t*+1_ is the conditional covariance between currency returns and home market portfolio returns, and $\gamma ^{c}_{1}$ corresponds to the price of currency risk. The model is estimated by quasi-maximum likelihood. During the estimation process for the first subsample, I encountered some convergence problems, which are a common issue of GARCH models when put into practice (see for example Silvennoinen and Teräsvirta (2009)), so I only present results for the full sample and the second subsample.

**Fig. 7:**
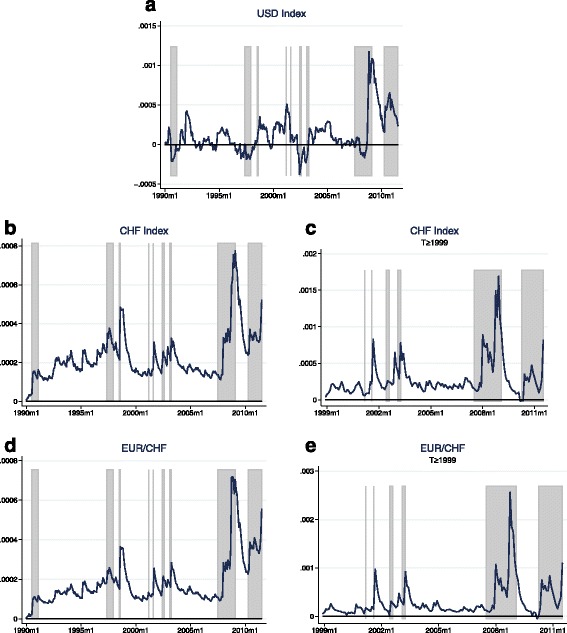
(**a**-**e**) GARCH results—conditional covariance estimates. *Notes:* The estimates of the conditional covariance correspond to the fitted values of the DCC MGARCH-in-mean model for the case of no time variation in the price of risk. For details see section D.1.1 in the Additional file 1

The conditional covariances implied by the GARCH model are pictured in Fig. 7. In the case of the USD, its evolution is pretty comparable to the one estimated with instruments. In the case of the CHF, across both exchange rates and samples, it now looks much closer to what one would expect, with clear peaks in crisis episodes. Altogether, the estimates for the conditional covariance implied by the GARCH models seem to be more convincing than the ones calculated with instruments.

The other object of interest are the price of risk estimates (see the Table 9). They are all positive, even though insignificant. Overall, they are roughly comparable in magnitude to my three-step GMM estimates when using the zero-stage prediction to measure the expected exchange rate change (recall the values of the last and second to the last column in Table 7) and thus support this solution to the measurement error problem. Based on the second period GARCH estimates, the safety premium for the Swiss franc reflected in the EUR/CHF exchange rate would be 2.5% (on an annual basis) on average and reach its maximum of around 12.5% during the recent financial crisis^21^, thus values that are larger than the ones suggested by my GMM results from the“Second-stage regression results” section.

**Table 9:** GARCH - constant price of risk

	USD index	CHF index		EUR/CHF	
$xr^{c}_{t+1}$	*All*	*All*	*T*≥1999	*All*	*T*≥1999
*σ*_*c,m,t*+1_	6.150	3.758	10.168	2.208	3.482
	[6.038]	[10.560]	[6.896]	[11.767]	[3.192]
*Cons*	0.000	−0.000	−0.002	0.001	0.001
	[0.001]	[0.002]	[0.002]	[0.002]	[0.001]

*Notes:* This table reports the quasi-maximum likelihood estimates for the currency excess returns mean equation of the DCC MGARCH model for the case of no time variation in the price of risk: $xr^{c}_{t+1} = \gamma ^{c}_{0} + \gamma ^{c}_{1} \sigma _{c,m,t+1} + u^{c}_{t+1}$. $xr^{c}_{t+1}$ corresponds to the excess return of investing abroad, *σ*_*c,m,t*+1_ is the conditional covariance between currency returns and home market portfolio returns, and $\gamma ^{c}_{1}$ corresponds to the price of currency risk. For details and the complete results table see the Additional file 1. Robust standard errors are reported in square brackets

***, **, * denote significance levels of 1, 5, and 10%, respectively

Altogether, however, also this GARCH model finds only weak evidence that investors are rewarded for their exposure to currency risk, which is consistent with earlier GARCH literature. De Santis and Gérard (1998) estimate a BEKK GARCH model to find the magnitude of the premium for currency risk based on the international CAPM and only obtain insignificant results when estimating constant prices of risk^22^. So, for completeness, I also let the price of risk in my GARCH specification change through time. The according results are presented and discussed in detail in the Additional file 1. Overall, the performance of the models is slightly better with a time-varying price of risk, which, however, goes with a higher complexity of the models, measured by the number of parameters. Various information criteria suggest going with the constant price of risk version. Thus, restricting the price of risk to be constant seems still to be justified.

## Conclusions

In this paper, I show that the three-step GMM approach that Maggiori (2013) uses to calculate the USD safety premium does not work for the CHF. The price of risk estimates take unrealistic, negative values. One guess why this is the case is his choice of how to measure the expected exchange rate change. Taking the actual ex-post exchange rate to measure the expected exchange rate, the dependent variable incorporates the prediction error, which in the case of exchange rates is likely to be large as they are hard to forecast in the short term. This measurement error in the dependent variable is a potential source of imprecision. I try two alternative ways to proxy the expected exchange rate change. The results get more plausible, especially when using the predictions of an augmented Fama regression, but are still not fully convincing due to the poor performance of the instruments. A maximum likelihood-estimated GARCH model seems to be a better choice for estimating the conditional safety premium as it allows to estimate the model elegantly in one single step. Furthermore, there is no need for finding good instruments.

Once a potential structural break in the relationship between Swiss franc exchange rate returns and equity returns is taken into account, the above findings provide evidence that the conditional international CAPM can help to explain the dynamics of the CHF returns versus a basket of currencies and the Euro in particular. My results reveal that the conditional covariance between stock market shocks and the Swiss franc exchange rate varies significantly through time, is almost always positive and reaches its peaks during crisis times, confirming that investors expect it to appreciate after bad shocks. There is some evidence for the price of currency risk being time-varying, but at the same time, this evidence still justifies to go with a constant price of risk. Finally, my CHF safety premium estimates vary depending on which estimation strategy is used, being rather low when estimated with the three-step GMM approach, a finding that I mainly attribute to the weak instruments. The GARCH approaches finds slightly higher values, suggesting that the CHF safety premium was on average equal to around 2.5% (on an annual basis) between early 1999 and mid-2011, and around 4.5% with peaks of up to 12.5% during the recent financial crisis. Overall, these findings support the view of the CHF acting as a safe haven during crises.

## Appendix

**Table 10:** List of countries

USD index	CHF index	*Selected euro area countries*
Selected Euro Area Countries	Selected Euro Area Countries	Austria
Australia	United States	Belgium-Luxembourg
Canada	United Kingdom	Finland
Denmark	China (including Hong Kong)	France
Hong Kong	Japan	Germany
Israel		Greece
Japan		Ireland
New Zealand		Italy
Norway		Netherlands
Singapore		Portugal
Sweden		Spain
Switzerland		
United Kingdom		

**Table 11:** Episodes of crisis (stock market volatility shocks)

Event	Period
Gulf War I	August 1990–March 1991
Asian Crisis	June 1997–January 1998
Russian Crisis, LTCM Default	August–September 1998
Dotcom Bust*	April 2001
9/11 Terrorist Attacks	September 2001
Worldcom, Enron Bankruptcy	July–September 2002
Gulf War II	March–May 2003
Credit Crunch, Lehman Default	August 2007–March 2009, October 2008
Greek Government-Debt Crisis*	May 2010–end of sample

All episodes except the ones marked by * are taken from Bloom (2009). Bloom identifies periods of major stock market volatility shocks by analysing the deviations of a stock market volatility series from its detrended mean. I partially extend the length of these periods as the events leading to this increased volatility in stock markets already started earlier and lasted longer than indicated by Bloom and because there is evidence in my time series of extensive market reaction. I followed Maggiori (2013) in adding the event of the dotcom bust, and finally included the the recent Greek government-debt crisis

## Additional file

Additional file 1 Online Appendix to “The Swiss Franc Safety Premium” (available on the author’s personal website). (PDF 410 kb)

## Footnotes

1 Note that the USD Index is a market-capitalization weighted index, while the CHF Index is a trade-weighted index. For justification (also for the choice of the stock market returns) and further details, see “Data” section.

2 All subsequent findings also hold if *D*_*t*+1_ is defined as , instead.

3 See section B in the Additional file 1 for equivalent figures with *D*_*t*+1_ defined as . The pattern is very similar.

4 Although deeper analysis suggests that there could be other possible break dates, I decided to split the sample at January 1999 as this date stands for an exogenous change in international monetary conditions and as it gives me two roughly equally sized subsamples.

5 These findings are robust to various measures of the pre-Euro EUR/CHF exchange rate. The one used in this paper is a trade-weighted index considering a sample of 11 future Euro area countries. The asymmetry pattern, however, is also present when the EUR/CHF exchange rate before 1999 only considers the Deutsche Mark.

6 See section B in the Additional file 1 for an equivalent table with the correlation depending on whether the equity returns are below or above 0. The pattern is very similar.

7 Duffee (2005) refers to the first step as the zero-stage regression as the two following steps are the typical GMM-IV-setup first- and second-stage regressions.

8 For more details on the exact estimation procedure, see Maggiori (2013).

9 This choice of the sample length is mainly driven by limited data availability.

10 Even tough the years 2012 and 2013 are not part the sample I analyze, I include them when constructing my market capitalization weights as they might act as a counterbalance to the exceptional years of 2008 to 2010.

11 Results obtained when using the same stock market index and the same time-span as Maggiori (2013) are provided in section C.8 in the Additional file 1.

12 Typically, the interest differential is defined the other way round (home minus foreign). This is why, usually, they rather say that we should expect a coefficient of 1.

13 For completeness, Additional file 1: Section C.1 provides first-stage and second-stage results obtained when the lagged exchange rate change is used as an instrument, but not as a regressor in the Fama regression.

14 Maggiori uses an iterative GMM estimator, which Ferson and Foerster (1994) find to have the better properties in finite sample estimations. In my case, however, this estimator produces results that lie far from the ones produced by the simple or by the two-step GMM estimator, raising some doubts about its validity (see Cochrane (1996)).

15 Results obtained when using the same stock market index, the same time-span and iterated GMM as Maggiori (2013) does are presented in section C.8 in the Additional file 1.

16 A Monte Carlo exercise suggests that the estimates of the standard deviation based on the asymptotic GMM formula are reasonable.

17 See the bottom right figure in Fig. 6 and the last column in the lower part of Table 7. Hence, for the safety premium at the peak in beginning 2009, for example, calculate (1−0.001+0.0012∗3.211)^12^−1.

18 See the bottom right figure in Figure A.3 and the last column in the lower part of Table A.7.

19 Furthermore, there is evidence that risk aversion varies through time. See, for example, Bliss and Panigirtzoglou (2004), Bekaert and Hoerova (2016) and Kim et al. (2010).

20 Dynamic Conditional Correlation model by Engle (2002).

21 See the bottom right figure in Fig. 7 and the last column in Table 9.

22 While the GARCH specifications BEKK by Engle and Krone (1995) and DCC are shown to produce very similar results (see Caporin and McAleer (2008, 2012)), the DCC model is computationally more attractive.
