# Supplementary material for: The Swiss franc safety premium
Source: Swiss J Econ Stat. 2018 Apr 17;154(1):13. doi: 10.1186/s41937-017-0014-7 (PMC6214287; doi:10.1186/s41937-017-0014-7)
Supplement: Supplementary file 1 — Online Appendix to “The Swiss Franc Safety Premium” (available on the author’s personal website). (PDF 410 kb) [file 41937_2017_14_MOESM1_ESM.pdf]

Online Appendix to “The Swiss Franc Safety Premium”

Jessica Leutert

Not for Publication. May 2017.

A Log-Linearization

$$0 = E_t\left[M_{t+1}\left(R^*_{t+1}\frac{\mathcal{E}_{t+1}}{\mathcal{E}_t} - R_{t+1}\right)\right]$$

$$\Leftrightarrow E_t\left[M_{t+1}R_{t+1}\right] = E_t\left[M_{t+1}\left(R^*_{t+1}\frac{\mathcal{E}_{t+1}}{\mathcal{E}_t}\right)\right]$$

Assume that all variables are jointly log-normally distributed and use the fact that z being normally distributed means that $E[e^z] = e^{E[z]+(1/2)\sigma^2[z]}$. Then:

$$E_t\left[M_{t+1}R_{t+1}\right] = E_t\left[M_{t+1}\left(R^*_{t+1}\frac{\mathcal{E}_{t+1}}{\mathcal{E}_t}\right)\right]$$

$$\Leftrightarrow E_t\left[e^{log(M_{t+1}R_{t+1})}\right] = E_t\left[e^{log\left(M_{t+1}\left(R^*_{t+1}(\mathcal{E}_{t+1}/\mathcal{E}_t)\right)\right)}\right]$$

$$\Leftrightarrow e^{E_t[log(M_{t+1}R_{t+1})]+(1/2)\sigma^2_t[log(M_{t+1}R_{t+1})]} = e^{E_t\left[log\left(M_{t+1}\left(R^*_{t+1}(\mathcal{E}_{t+1}/\mathcal{E}_t)\right)\right)\right]+(1/2)\sigma^2_t\left[log\left(M_{t+1}\left(R^*_{t+1}(\mathcal{E}_{t+1}/\mathcal{E}_t)\right)\right)\right]}$$

$$\Leftrightarrow E_t[m_{t+1}] + E_t[r_{t+1}] + \frac{1}{2}Var_t[m_{t+1} + r_{t+1}] = E_t[m_{t+1}] + E_t[r^*_{t+1}] + E_t[\triangle e_{t+1}] + \frac{1}{2}Var_t[m_{t+1} + r^*_{t+1} + \triangle e_{t+1}]$$

$$\Leftrightarrow E_t[r_{t+1}] + \frac{1}{2}\left(Var_t[m_{t+1}] + Var_t[r_{t+1}] + 2Cov_t[m_{t+1}, r_{t+1}]\right) = E_t[r^*_{t+1}] + E_t[\triangle e_{t+1}] + \frac{1}{2}\left(Var_t[m_{t+1}] + Var_t[r^*_{t+1} + \triangle e_{t+1}] + 2Cov_t[m_{t+1}, r^*_{t+1} + \triangle e_{t+1}]\right)$$

$$\Leftrightarrow E_t[r_{t+1}] + \frac{1}{2}Var_t[r_{t+1}] + Cov_t[m_{t+1}, r_{t+1}] = E_t[r^*_{t+1}] + E_t[\triangle e_{t+1}] + \frac{1}{2}Var_t[r^*_{t+1} + \triangle e_{t+1}] + Cov_t[m_{t+1}, r^*_{t+1}] + Cov_t[m_{t+1}, \triangle e_{t+1}]$$

$$\Leftrightarrow E_t\left[r^*_{t+1} + \triangle e_{t+1} - r_{t+1}\right] + \frac{1}{2}Var_t\left(r^*_{t+1} + \triangle e_{t+1}\right) - \frac{1}{2}Var_t\left(r_{t+1}\right) = -Cov_t\left(m_{t+1}, r^*_{t+1}\right) + Cov_t\left(m_{t+1}, r_{t+1}\right) - Cov_t\left(m_{t+1}, \triangle e_{t+1}\right)$$

The complete market assumption implies that the exchange rate change is equal to the difference between the (log) foreign and home discount factors: $\triangle e_{t+1} = m^*_{t+1} - m_{t+1}$. Using this finally

yields:

$$\Rightarrow E_t\left[r^*_{t+1}+\triangle e_{t+1}-r_{t+1}\right]+\frac{1}{2}Var_t\left(r^*_{t+1}+\triangle e_{t+1}\right)-\frac{1}{2}Var_t\left(r_{t+1}\right)=$$
$$-Cov_t\left(m^*_{t+1},r^*_{t+1}\right)+Cov_t\left(m_{t+1},r_{t+1}\right)+Cov_t\left(r^*_{t+1},\triangle e_{t+1}\right)-Cov_t\left(m_{t+1},\triangle e_{t+1}\right)$$

B Descriptive Evidence when Defining D_{t+1} as $\mathbb{1}_{r^{\omega}_{t+1}<0}$

Figure A.1: Scatterplots and fitted regression line for $D_{t+1} \equiv \mathbb{1}_{r^{\omega}_{t+1}<0}$

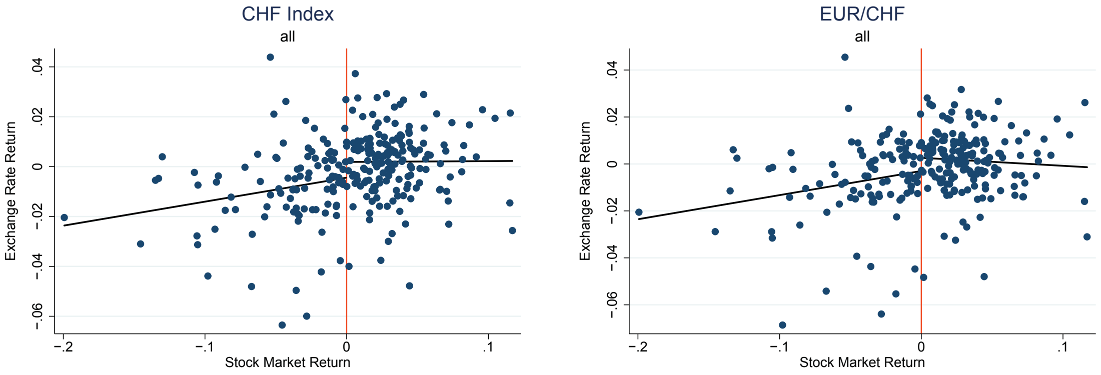

Notes: These scatterplots show the relationship between monthly domestic stock market returns and exchange rate returns (end of period values) for the time period January 1990 to August 2011. Domestic stock market returns are calculated from the S&P 500 Index in case of the USD exchange rate index and the SPI for the two CHF exchange rates.

Table A.3: Correlation Table

	$Corr(r^{\omega}_{t+1}, \triangle e_{t+1})$ for $r^{\omega}_{t+1} < 0$	$Corr(r^{\omega}_{t+1}, \triangle e_{t+1})$ for $r^{\omega}_{t+1} \geq 0$
**USD Index**		
all	0.28***	0.26***
**CHF Index**		
all	0.21**	0.01
$T < 1999$	0.35**	-0.09
$T \geq 1999$	0.13	0.16
**EUR/CHF**		
all	0.22**	-0.06
$T < 1999$	0.37**	-0.17
$T \geq 1999$	0.17	0.13

Notes: Correlation between monthly local stock market returns and exchange rate returns (end of period values) for the time period January 1990 to August 2011, depending on whether stock returns are below or above 0. Local stock market returns are calculated from the S&P 500 Index in case of the USD exchange rate index and the SPI for the two CHF exchange rates. ***, **, and * denote significance levels of 1, 5, and 10%, respectively, based on a t-test.

C Robustness

For completeness, I provide here the results for the subperiods for (1) when the exchange rate change is included in the set of instruments and (2) when the set of instruments is optimized. Furthermore, second stage regression results are presented for (3) when the local stock market index is replaced by a global stock market index and (4) when the global financial crisis of 2007-2008 and the Great Recession are excluded from the sample. For robustness, finally, results are presented for (5) a financially weighted CHF index, (6) a sample beginning in 1975 and (7) a sample beginning in 1987.

C.1 Set of Instruments Including the Exchange Rate

Here, I exclude the lagged exchange rate change as regressor in the zero-stage regression (and hence use the predictions of the original Fama model to construct the dependent variable in the second stage regressions). In return, I include the lagged exchange rate change in the set of instruments for the conditional covariance.

Figure A.2: Predicted Conditional Covariance - Total Set of Instruments

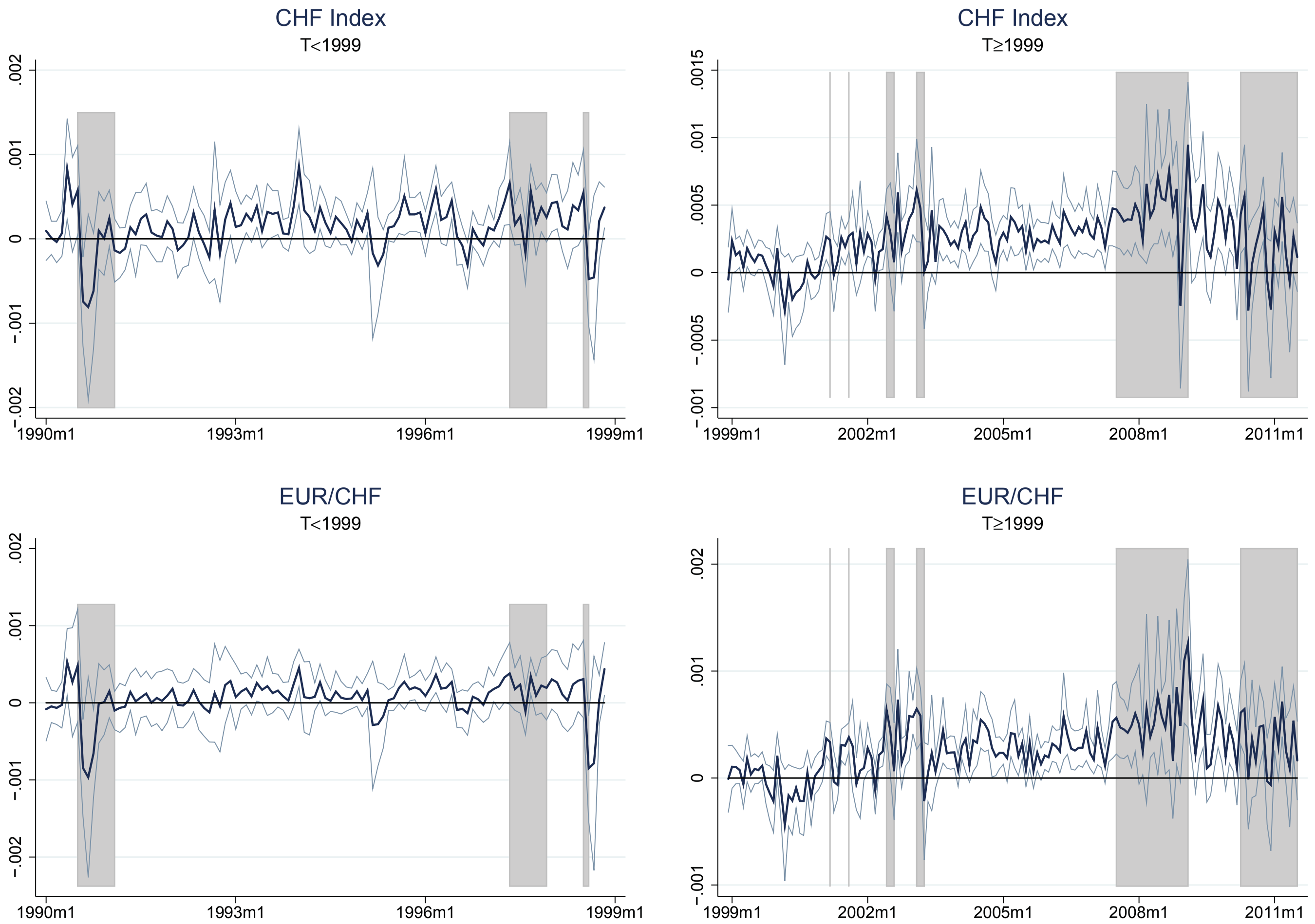

Notes: The estimates of the conditional covariance correspond to the fitted value of the first stage regression: $\widehat{Cov_t}\left(r^{\omega}_{t+1}, \triangle e_{t+1}\right) = \hat{\alpha}_Z Z_t$ (see equation (9)). In this first stage regression, the ex-post covariance obtained from the zero stage regressions is regressed on a set of instruments. The set of instruments Z_t consists of a constant, the dividend-price ratio, the lagged equity return, the lagged exchange rate change, plus a measure for the lagged equity return variance, exchange rate return variance, and their covariance. The zero stage regressions are estimated for each subsample separately. The first subsample (January 1990 to December 1998) consists of 107 observations and the second subsample (January 1999 to August 2011) consists of 152 observations. The two thin lines represent the 95% confidence band and are based on a two sided t-statistic with Newey-West estimates of the standard errors.

Table A.4: First Stage Regression - Total Set of Instruments

	CHF Index			EUR/CHF	
$\widetilde{Cov}_t\left(r^{\omega}_{t+1}, \triangle e_{t+1}\right)$	$T < 1999$	$T \geq 1999$		$T < 1999$	$T \geq 1999$
dp_t	-0.000	0.001**		-0.000	0.001**
[0.001]	[0.000]		[0.001]	[0.000]	
r^{ω}_t	0.002	-0.003**		0.001	-0.005**
	[0.001]	[0.002]		[0.001]	[0.002]
$\triangle e_t$	-0.006	0.007**		-0.002	0.007***
	[0.005]	[0.003]		[0.005]	[0.003]
$var^{'e}_t$	-0.088	-0.154		-0.100	-0.052
	[0.111]	[0.096]		[0.105]	[0.128]
$var^{'r}_t$	0.002	-0.019		-0.006	-0.018
	[0.012]	[0.016]		[0.013]	[0.018]
$cov^{'}_t$	-0.162**	0.045		-0.131	-0.018
	[0.066]	[0.071]		[0.082]	[0.082]
$Cons$	-0.001	0.003***		-0.001	0.004**
	[0.002]	[0.001]		[0.002]	[0.002]
R^2	0.105	0.102		0.095	0.122
F-statistic	3.863	3.140		6.298	5.803
χ^2-statistic	23.178	18.840		37.788	34.818
p-value (χ^2-stat.)	0.001	0.004		0.000	0.000

Notes: This table reports the results of the first stage regression, which regresses the ex-post covariance obtained from the zero stage regressions on a set of instruments (see equation (8)). This set of instruments Z_t consists of a constant, the dividend-price ratio, the lagged equity return, the lagged exchange rate change, plus a measure for the lagged equity return variance, exchange rate return variance, and their covariance. The parameters are estimated by OLS using Newey-West standard errors with maximum lag order set equal to $T^{1/2}$. The F-statistic and the Wald χ^2 test (plus the p-value for the Wald χ^2 test) are reported for the null hypothesis that all coefficients, except the constant, are jointly zero. The model is estimated separately for each subsample. The first subsample (January 1990 to December 1998) consists of 107 observations and the second subsample (January 1999 to August 2011) consists of 152 observations. The standard errors are reported in square brackets. ***, **, and * denote significance levels of 1, 5, and 10%, respectively.

Table A.5: Second Stage Regression - Total Set of Instruments.

Subperiod 1

$r^*_{f,t+1} + E_t[\triangle e_{t+1}] - r_{f,t+1}$	$E_t[\triangle e_{t+1}] = \triangle e_{t+1}$		$E_t[\triangle e_{t+1}] = 0$		$E_t[\triangle e_{t+1}] = \widehat{E_t[\triangle e_{t+1}]}$	
$+\frac{1}{2}\widetilde{Var}_t\,(\triangle e_{t+1})$	CHF Index	EUR/CHF	CHF Index	EUR/CHF	CHF Index	EUR/CHF
$\widetilde{Cov}_t\left(r^{\omega}_{t+1}, \triangle e_{t+1}\right)$	-6.059	-6.087**	0.541***	0.748**	2.811***	2.498**
	[4.896]	[3.600]	[0.176]	[0.346]	[1.022]	[1.158]
Cons	0.001	0.002*	0.002***	0.002***	0.001	0.001***
	[0.002]	[0.001]	[0.000]	[0.000]	[0.001]	[0.000]
J-statistic	3.923	2.930	6.408	4.551	6.846	4.555
p-value	0.561	0.711	0.269	0.473	0.232	0.473

Subperiod 2

$r^*_{f,t+1} + E_t[\triangle e_{t+1}] - r_{f,t+1}$	$E_t[\triangle e_{t+1}] = \triangle e_{t+1}$		$E_t[\triangle e_{t+1}] = 0$		$E_t[\triangle e_{t+1}] = \widehat{E_t[\triangle e_{t+1}]}$	
$+\frac{1}{2}\widetilde{Var}_t\,(\triangle e_{t+1})$	CHF Index	EUR/CHF	CHF Index	EUR/CHF	CHF Index	EUR/CHF
$\widetilde{Cov}_t\left(r^{\omega}_{t+1}, \triangle e_{t+1}\right)$	-5.024*	-5.627	0.266**	0.415***	1.054*	1.153**
	[3.791]	[4.839]	[0.123]	[0.115]	[0.696]	[0.620]
Cons	0.001	0.001	0.001***	0.001***	-0.000	-0.001**
	[0.001]	[0.001]	[0.000]	[0.000]	[0.001]	[0.000]
J-statistic	3.501	4.857	4.044	4.630	4.161	4.505
p-value	0.623	0.434	0.543	0.463	0.526	0.479

Notes: This table reports the results of the second stage regression for the case of no time variation in the price of risk (see equation (13)). The dependent variable is the CHF safety premium, defined as the expected excess return of investing in the foreign risk-free asset by shorting the home risk-free asset. The expected exchange rate change used to calculate this expected excess return is proxied first by the actual exchange rate change, then by zero, and finally by the fitted value of the zero stage regression. The regressors are a constant and the estimate of the conditional covariance between stock returns and exchange rate changes from the first stage regression estimated with the complete set of instruments. This consists of a constant, the dividend-price ratio, the lagged equity return, the lagged exchange rate change, plus a measure for the lagged equity return variance, exchange rate return variance, and their covariance. The second stage regression is estimated jointly with the zero stage regression by GMM which allows the standard errors of the second stage regression to incorporate not only the uncertainty deriving from the first-stage regression, but also the one from the zero stage regression. The standard errors are based on the Newey-West estimate of the covariance matrix with maximum lag order set equal to $T^{1/2}$. The J-statistic (Hansen, 1982) plus the according p-value are reported for the null hypothesis that the model is well-specified and the moment conditions do hold. The model is estimated for each subsample separately. The first subsample (January 1990 to December 1998) consists of 107 observations and the second subsample (January 1999 to August 2011) consists of 152 observations. The standard errors are reported in square brackets. ***, **, and * denote significance levels of 1, 5, and 10%, respectively.

C.2 Optimal Set of Instruments

Here, the instruments for each model are chosen such as to maximize the F-statistic in the first stage regression.

Table A.6: First Stage Regression - "Optimal" Set of Instruments

	CHF Index		EUR/CHF	
$\widetilde{Cov}_t\left(r^{\omega}_{t+1}, \triangle e_{t+1}\right)$	$T < 1999$	$T \geq 1999$	$T < 1999$	$T \geq 1999$
dp_t		0.001***		0.001***
		[0.000]		[0.000]
r^{ω}_t	0.002	-0.002	0.002	-0.003**
	[0.001]	[0.001]	[0.001]	[0.001]
$var^{'e}_t$		-0.157**	-0.114	
		[0.069]	[0.116]	
$var^{'r}_t$			-0.004	
			[0.010]	
$cov^{'}_t$	-0.148***		-0.135*	
	[0.051]		[0.077]	
$Cons$	0.000**	0.003***	0.000	0.003***
	[0.000]	[0.001]	[0.000]	[0.001]
R^2	0.077	0.075	0.089	0.082
F-statistic	11.580	2.750	7.978	6.607
χ^2-statistic	23.160	8.250	31.912	13.214
p-value (χ^2-stat.)	0.000	0.041	0.000	0.001

Notes: This table reports the results of the first stage regression, which regresses the ex-post covariance obtained from the zero stage regressions on an "optimal" set of instruments (see equation (8)). Instruments are selected such as to maximize the F-statistic. The set of possible instruments Z_t consists of a constant, the dividend-price ratio, the lagged equity return, plus a measure for the lagged equity return variance, exchange rate return variance, and their covariance. The parameters are estimated by OLS using Newey-West standard errors with maximum lag order set equal to $T^{1/2}$. The F-statistic and the Wald χ^2 test (plus the p-value for the Wald χ^2 test) are reported for the null hypothesis that all coefficients, except the constant, are jointly zero. The model is estimated separately for each subsample. The first subsample (January 1990 to December 1998) consists of 107 observations and the second subsample (January 1999 to August 2011) consists of 152 observations. The standard errors are reported in square brackets. ***, **, and * denote significance levels of 1, 5, and 10%, respectively.

Figure A.3: Predicted Conditional Covariance - "Optimal" Set of Instruments

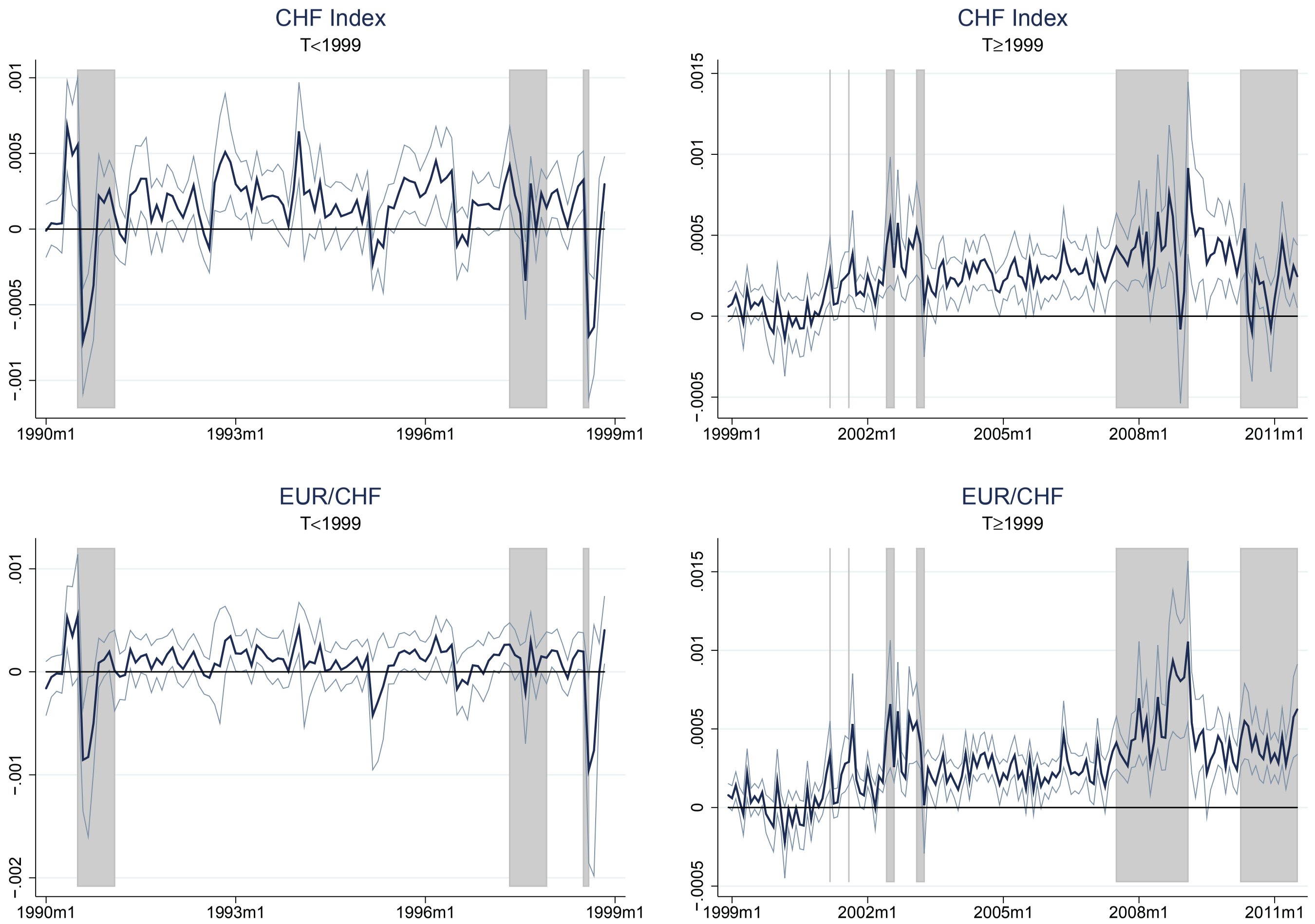

Notes: The estimates of the conditional covariance correspond to the fitted value of the first stage regression: $\widehat{Cov_t}\left(r^{\omega}_{t+1}, \triangle e_{t+1}\right) = \hat{\alpha}_Z Z_t$ (see equation (9)). In this first stage regression, the ex-post covariance obtained from the zero stage regressions is regressed on the "optimal" set of instruments. The possible set of instruments Z_t consists of a constant, the dividend-price ratio, the lagged equity return, the lagged exchange rate change, plus a measure for the lagged equity return variance, exchange rate return variance, and their covariance. The zero stage regressions are estimated for each subsample separately. The first subsample (January 1990 to December 1998) consists of 107 observations and the second subsample (January 1999 to August 2011) consists of 152 observations. The two thin lines represent the 95% confidence band and are based on a two sided t-statistic with Newey-West estimates of the standard errors.

Table A.7: Second Stage Regression - "Optimal" Set of Instruments.

Subperiod 1

$r^*_{f,t+1} + E_t[\triangle e_{t+1}] - r_{f,t+1} + \frac{1}{2}\widetilde{Var}_t(\triangle e_{t+1})$	$E_t[\triangle e_{t+1}] = \triangle e_{t+1}$		$E_t[\triangle e_{t+1}] = 0$		$E_t[\triangle e_{t+1}] = E_t[\widehat{\triangle e_{t+1}}]$	
	CHF Index	EUR/CHF	CHF Index	EUR/CHF	CHF Index	EUR/CHF
$\widetilde{Cov}_t(r^{\omega}_{t+1}, \triangle e_{t+1})$	0.010	-7.195*	0.441	0.672**	3.401*	2.272**
	[6.472]	[4.553]	[0.352]	[0.355]	[2.268]	[1.186]
Cons	0.000	0.002*	0.002***	0.002***	0.001	0.001***
	[0.002]	[0.001]	[0.000]	[0.000]	[0.001]	[0.000]
J-statistic	1.957	2.775	4.420	4.625	4.467	4.705
p-value	0.162	0.428	0.036	0.201	0.035	0.195

Subperiod 2

$r^*_{f,t+1} + E_t[\triangle e_{t+1}] - r_{f,t+1} + \frac{1}{2}\widetilde{Var}_t(\triangle e_{t+1})$	$E_t[\triangle e_{t+1}] = \triangle e_{t+1}$		$E_t[\triangle e_{t+1}] = 0$		$E_t[\triangle e_{t+1}] = E_t[\widehat{\triangle e_{t+1}}]$	
	CHF Index	EUR/CHF	CHF Index	EUR/CHF	CHF Index	EUR/CHF
$\widetilde{Cov}_t(r^{\omega}_{t+1}, \triangle e_{t+1})$	-1.485	-0.998	0.284	0.730***	3.635	6.151***
	[4.405]	[4.596]	[0.267]	[0.225]	[3.519]	[1.856]
Cons	0.000	0.000	0.001***	0.001***	-0.001**	-0.002***
	[0.001]	[0.001]	[0.000]	[0.000]	[0.001]	[0.000]
J-statistic	1.953	0.548	1.236	1.372	4.853	5.655
p-value	0.377	0.459	0.539	0.241	0.088	0.017

Notes: This table reports the results of the second stage regression for the case of no time variation in the price of risk (see equation (13)). The dependent variable is the CHF safety premium, defined as the expected excess return of investing in the foreign risk-free asset by shorting the home risk-free asset. The expected exchange rate change used to calculate this expected excess return is proxied first by the actual exchange rate change, then by zero, and finally by the fitted value of the zero stage regression. The regressors are a constant and the estimate of the conditional covariance between stock returns and exchange rate changes from the first stage regression estimated with the "optimal" set of instruments. The possible set of instruments Z_t consists of a constant, the dividend-price ratio, the lagged equity return, plus a measure for the lagged equity return variance, exchange rate return variance, and their covariance. The second stage regression is estimated jointly with the zero stage regression by GMM which allows the standard errors of the second stage regression to incorporate not only the uncertainty deriving from the first-stage regression, but also the one from the zero stage regression. The standard errors are based on the Newey-West estimate of the covariance matrix with maximum lag order set equal to $T^{1/2}$. The J-statistic (Hansen, 1982) plus the according p-value are reported for the null hypothesis that the model is well-specified and the moment conditions do hold. The model is estimated for each subsample separately. The first subsample (January 1990 to December 1998) consists of 107 observations and the second subsample (January 1999 to August 2011) consists of 152 observations. The standard errors are reported in square brackets. ***, **, and * denote significance levels of 1, 5, and 10%, respectively.

C.3 Global Stock Market Index

This section reports the first and second stage results when the MSCI World index instead of the local stock market indices is taken to measure the investor's benchmark return.

Unlike in the benchmark case with the local stock market indices, one has now to be aware of the fact that the covariance between the exchange rate and the global stock market index converted into the respective currency incorporates direct exchange rate effects. For illustration, think of a situation where the local currency appreciates, while the global stock market index remains stable. In that case, the covariance between the exchange rate and the global stock market index converted into the local currency is positive even tough the value of the global stock market index has not changed. In the case of a safe currency, this implies that the covariance will tend to be overestimated.

While the price of risk estimates now take less extreme negative values when using the actual ex-post exchange rate change to measure the expected exchange rate change, the results in Tables A.8 and A.9 still support the main findings. With the use of the alternative measures for the expected exchange rate change, the standard deviations of the price of risk coefficients become smaller. When the expected exchange rate change is proxied by the zero stage regression, the results using global stock market returns get better (relative to the results using local stock market returns) for the full sample, while they get worse for the first subsample and are relatively stable for the second subsample. When considering the full sample and the second subsample, in three out of four cases the price of risk estimate for the CHF exchange rates lies between 2 and 3, i.e. values that are close to the ones found in the benchmark case. Overall, this robustness test supports the above finding that proxying the expected exchange rate change by the prediction of the zero stage regression yields more realistic and reliable estimations of the price of risk as compared to measuring the expected exchange rate change by the actual ex-post exchange rate.

Figure A.4: Predicted Conditional Covariance - Global Stock Market Index

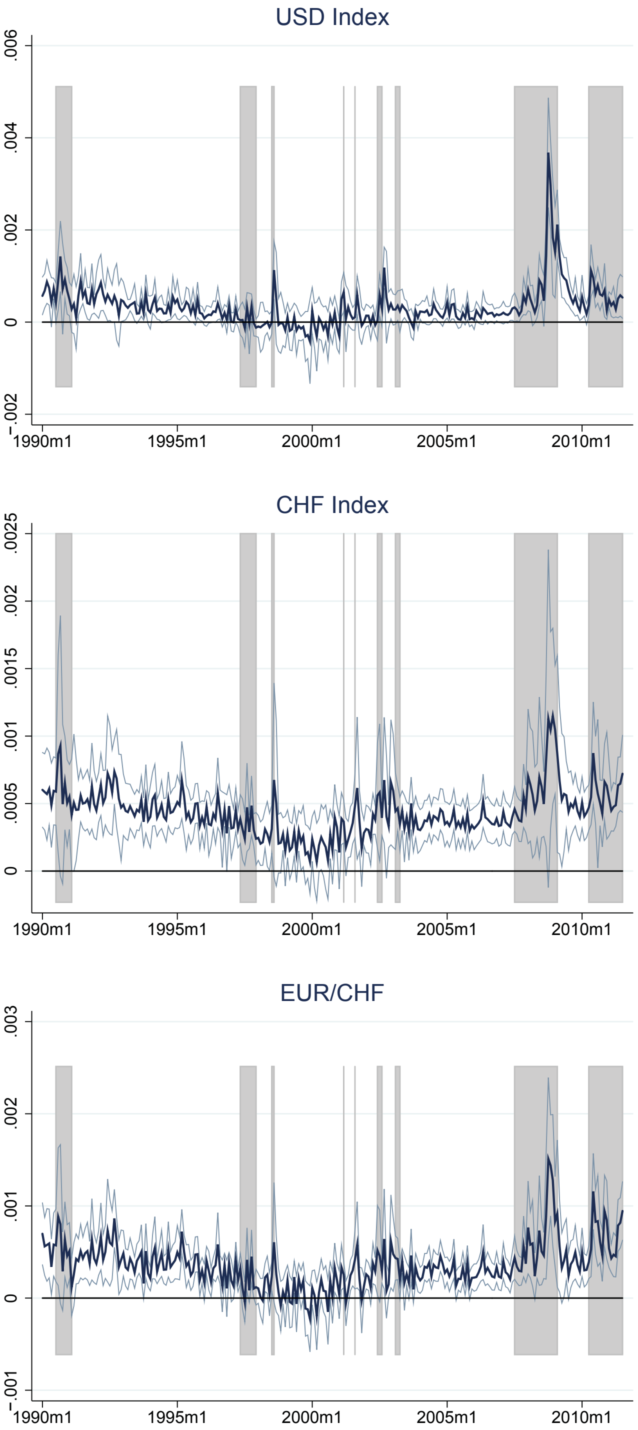

Notes: The estimates of the conditional covariance correspond to the fitted value of the first stage regression: $\widehat{Cov_t}\left(r^{\omega}_{t+1}, \triangle e_{t+1}\right) = \hat{\alpha}_Z Z_t$ (see equation (9)). In this first stage regression, the ex-post covariance obtained from the zero stage regressions is regressed on a set of instruments. The set of instruments Z_t consists of a constant, the dividend-price ratio, the lagged equity return, plus a measure for the lagged equity return variance, exchange rate return variance, and their covariance. The two thin lines represent the 95% confidence band and are based on a two sided t-statistic with Newey-West estimates of the standard errors.

Figure A.5: Predicted Conditional Covariance - Global Stock Market Index

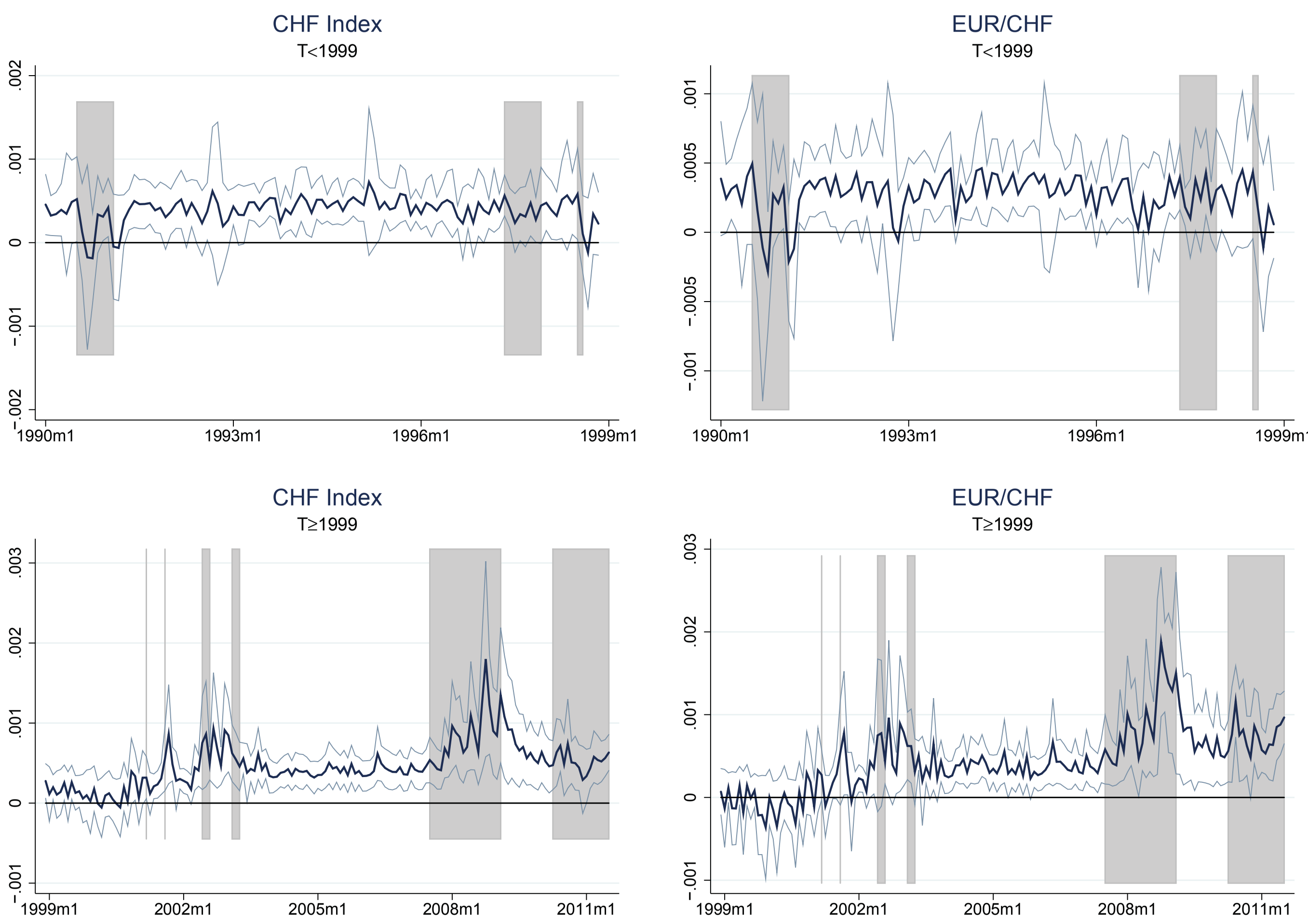

Notes: The estimates of the conditional covariance correspond to the fitted value of the first stage regression: $\widehat{Cov_t}\left(r^{\omega}_{t+1}, \triangle e_{t+1}\right) = \hat{\alpha}_Z Z_t$ (see equation (9)). In this first stage regression, the ex-post covariance obtained from the zero stage regressions is regressed on a set of instruments. The set of instruments Z_t consists of a constant, the dividend-price ratio, the lagged equity return, plus a measure for the lagged equity return variance, exchange rate return variance, and their covariance. The two thin lines represent the 95% confidence band and are based on a two sided t-statistic with Newey-West estimates of the standard errors.

Table A.8: Second Stage Regression - Global Stock Market Index - Full Sample

$r^*_{f,t+1} + E_t[\triangle e_{t+1}] - r_{f,t+1}$	$E_t[\triangle e_{t+1}] = \triangle e_{t+1}$			$E_t[\triangle e_{t+1}] = 0$			$E_t[\triangle e_{t+1}] = E_t[\widehat{\triangle e_{t+1}}]$		
$+\frac{1}{2}\widetilde{Var_t}(\triangle e_{t+1})$	USD Index	CHF Index	EUR/CHF	USD Index	CHF Index	EUR/CHF	USD Index	CHF Index	EUR/CHF
$\widetilde{Cov_t}(r^{\omega}_{t+1}, \triangle e_{t+1})$	5.561**	-5.112	1.967	1.393***	-0.833*	0.032	1.034	0.325	3.148***
	[3.088]	[5.234]	[2.484]	[0.515]	[0.645]	[0.292]	[1.023]	[1.743]	[0.922]
Cons	-0.000	0.002	-0.000	-0.000	0.002***	0.002***	0.001*	-0.000	-0.001**
	[0.002]	[0.002]	[0.001]	[0.000]	[0.000]	[0.000]	[0.001]	[0.001]	[0.000]
J-statistic	2.937	4.549	3.456	4.881	4.074	5.501	8.369	3.194	0.500
p-value	0.568	0.337	0.485	0.300	0.396	0.240	0.079	0.526	0.973

Notes: This table reports the results of the second stage regression for the case of no time variation in the price of risk (see equation (13)). The dependent variables are the USD and CHF safety premium, respectively, defined as the expected excess return of investing in the foreign risk-free asset by shorting the home risk-free asset. The expected exchange rate change used to calculate this expected excess return is proxied first by the actual exchange rate change, then by zero, and finally by the fitted value of the zero stage regression. The regressors are a constant and the estimate of the conditional covariance between stock returns and exchange rate changes from the first stage regression. The set of instruments Z_t consists of a constant, the dividend-price ratio, the lagged equity return, plus a measure for the lagged equity return variance, exchange rate return variance, and their covariance. The second stage regression is estimated jointly with the zero stage regression by GMM which allows the standard errors of the second stage regression to incorporate not only the uncertainty deriving from the first-stage regression, but also the one from the zero stage regression. The standard errors are based on the Newey-West estimate of the covariance matrix with maximum lag order set equal to $T^{1/2}$. The J-statistic (Hansen, 1982) plus the according p-value are reported for the null hypothesis that the model is well-specified and the moment conditions do hold. The number of observations is 259 for the full sample. The standard errors are reported in square brackets. ***, **, and * denote significance levels of 1, 5, and 10%, respectively.

Table A.9: Second Stage Regression - Global Stock Market Index

Subperiod 1

$r^*_{f,t+1} + E_t[\triangle e_{t+1}] - r_{f,t+1}$	$E_t[\triangle e_{t+1}] = \triangle e_{t+1}$		$E_t[\triangle e_{t+1}] = 0$		$E_t[\triangle e_{t+1}] = \widehat{E_t[\triangle e_{t+1}]}$	
$+\frac{1}{2}\widetilde{Var_t}(\triangle e_{t+1})$	CHF Index	EUR/CHF	CHF Index	EUR/CHF	CHF Index	EUR/CHF
$\widetilde{Cov_t}(r^{\omega}_{t+1}, \triangle e_{t+1})$	-0.213	-3.422	0.446	-0.164	0.636	-0.795
	[8.535]	[9.012]	[0.643]	[0.574]	[3.290]	[1.982]
Cons	0.000	0.002	0.002***	0.002***	0.001	0.001***
	[0.004]	[0.003]	[0.000]	[0.000]	[0.002]	[0.000]
J-statistic	4.013	3.159	6.406	5.112	7.010	5.489
p-value	0.404	0.532	0.171	0.276	0.135	0.241

Subperiod 2

$r^*_{f,t+1} + E_t[\triangle e_{t+1}] - r_{f,t+1}$	$E_t[\triangle e_{t+1}] = \triangle e_{t+1}$		$E_t[\triangle e_{t+1}] = 0$		$E_t[\triangle e_{t+1}] = \widehat{E_t[\triangle e_{t+1}]}$	
$+\frac{1}{2}\widetilde{Var_t}(\triangle e_{t+1})$	CHF Index	EUR/CHF	CHF Index	EUR/CHF	CHF Index	EUR/CHF
$\widetilde{Cov_t}(r^{\omega}_{t+1}, \triangle e_{t+1})$	-2.194	-0.187	0.196*	0.357***	2.419*	3.317***
	[2.672]	[2.203]	[0.137]	[0.100]	[1.545]	[0.883]
Cons	0.001	0.000	0.001***	0.001***	-0.002**	-0.002***
	[0.001]	[0.001]	[0.000]	[0.000]	[0.001]	[0.000]
J-statistic	3.609	4.280	3.852	3.922	7.011	5.744
p-value	0.461	0.369	0.426	0.417	0.135	0.219

Notes: This table reports the results of the second stage regression for the case of no time variation in the price of risk (see equation (13)). The dependent variables are the USD and CHF safety premium, respectively, defined as the expected excess return of investing in the foreign risk-free asset by shorting the home risk-free asset. The expected exchange rate change used to calculate this expected excess return is proxied first by the actual exchange rate change, then by zero, and finally by the fitted value of the zero stage regression. The regressors are a constant and the estimate of the conditional covariance between stock returns and exchange rate changes from the first stage regression. The set of instruments Z_t consists of a constant, the dividend-price ratio, the lagged equity return, plus a measure for the lagged equity return variance, exchange rate return variance, and their covariance. The second stage regression is estimated jointly with the zero stage regression by GMM which allows the standard errors of the second stage regression to incorporate not only the uncertainty deriving from the first-stage regression, but also the one from the zero stage regression. The standard errors are based on the Newey-West estimate of the covariance matrix with maximum lag order set equal to $T^{1/2}$. The J-statistic (Hansen, 1982) plus the according p-value are reported for the null hypothesis that the model is well-specified and the moment conditions do hold. The model is estimated for each subsample separately. The first subsample (January 1990 to December 1998) consists of 107 observations and the second subsample (January 1999 to August 2011) consists of 152 observations. The standard errors are reported in square brackets. ***, **, and * denote significance levels of 1, 5, and 10%, respectively.

C.4 Exclusion of Global Financial Crisis and Great Recession

This section presents first and second stage results when the period of the global financial crisis of 2007-2008 and the Great Recession are excluded from the sample. It reports the results of the second stage regression for the full sample and the second subsample when these samples are cut after July 2007. Table A.10 suggests that the highly negative values for the price of risk (recall Tables 6 and 7) when measuring the expected exchange rate change by the actual ex-post exchange rate change might at least partly be caused by the inclusion of the recent crisis episode. While for the full sample, the coefficients are still significantly negative, in the second subperiod they now are significantly positive. Again, the standard deviations of the price of risk coefficients are lower when the expected exchange rate change is measured by the prediction of the zero stage regression rather than by the actual ex-post exchange rate change. Overall, also this robustness test supports the above finding that proxying the expected exchange rate change by the prediction of the zero stage regression yields at least as or even more realistic and reliable estimations of the price of risk as compared to measuring the expected exchange rate change by the actual ex-post exchange rate.

Figure A.6: Predicted Conditional Covariance - Exclusion of Global Financial Crisis and Great Recession

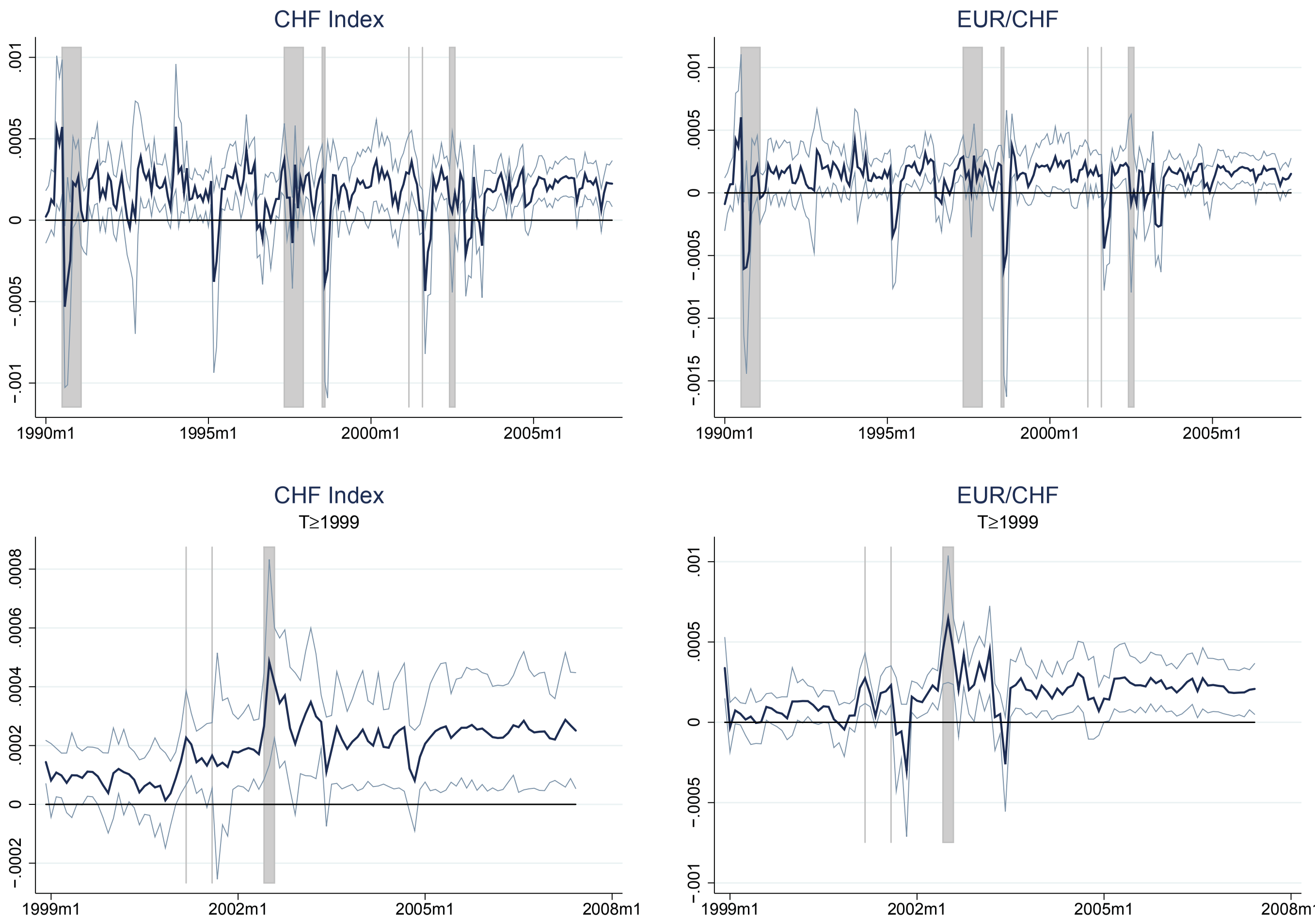

Notes: The estimates of the conditional covariance correspond to the fitted value of the first stage regression: $\widehat{Cov_t}\left(r^{\omega}_{t+1}, \triangle e_{t+1}\right) = \hat{\alpha}_Z Z_t$ (see equation (9)). In this first stage regression, the ex-post covariance obtained from the zero stage regressions is regressed on a set of instruments. The set of instruments Z_t consists of a constant, the dividend-price ratio, the lagged equity return, plus a measure for the lagged equity return variance, exchange rate return variance, and their covariance. The two thin lines represent the 95% confidence band and are based on a two sided t-statistic with Newey-West estimates of the standard errors.

Table A.10: Second Stage Regression - Exclusion of Global Financial Crisis and Great Recession

Full Sample

$r^*_{f,t+1} + E_t[\triangle e_{t+1}] - r_{f,t+1}$	$E_t[\triangle e_{t+1}] = \triangle e_{t+1}$		$E_t[\triangle e_{t+1}] = 0$		$E_t[\triangle e_{t+1}] = \widehat{E_t[\triangle e_{t+1}]}$	
$+\frac{1}{2}\widetilde{Var_t}(\triangle e_{t+1})$	CHF Index	EUR/CHF	CHF Index	EUR/CHF	CHF Index	EUR/CHF
$\widetilde{Cov_t}(r^{\omega}_{t+1}, \triangle e_{t+1})$	-7.657**	-8.673***	0.012	-0.067	0.009	-0.320
	[4.587]	[3.686]	[0.226]	[0.301]	[1.313]	[0.856]
Cons	0.002**	0.002***	0.002***	0.002***	0.000	0.001***
	[0.001]	[0.001]	[0.000]	[0.000]	[0.001]	[0.000]
J-statistic	3.322	3.583	7.275	7.786	7.677	7.311
p-value	0.505	0.465	0.122	0.100	0.104	0.120

Subperiod 2

$r^*_{f,t+1} + E_t[\triangle e_{t+1}] - r_{f,t+1}$	$E_t[\triangle e_{t+1}] = \triangle e_{t+1}$		$E_t[\triangle e_{t+1}] = 0$		$E_t[\triangle e_{t+1}] = \widehat{E_t[\triangle e_{t+1}]}$	
$+\frac{1}{2}\widetilde{Var_t}(\triangle e_{t+1})$	CHF Index	EUR/CHF	CHF Index	EUR/CHF	CHF Index	EUR/CHF
$\widetilde{Cov_t}(r^{\omega}_{t+1}, \triangle e_{t+1})$	12.069**	8.564**	0.970***	0.935***	5.777*	5.971***
	[6.795]	[4.375]	[0.390]	[0.227]	[3.720]	[1.925]
Cons	-0.001	0.001	0.001***	0.001***	0.001	0.000
	[0.002]	[0.001]	[0.000]	[0.000]	[0.001]	[0.000]
J-statistic	1.940	4.421	1.111	2.879	1.641	5.283
p-value	0.747	0.352	0.893	0.578	0.801	0.259

Notes: This table reports the results of the second stage regression for the case of no time variation in the price of risk (see equation (13)). The dependent variables are the USD and CHF safety premium, respectively, defined as the expected excess return of investing in the foreign risk-free asset by shorting the home risk-free asset. The expected exchange rate change used to calculate this expected excess return is proxied first by the actual exchange rate change, then by zero, and finally by the fitted value of the zero stage regression. The regressors are a constant and the estimate of the conditional covariance between stock returns and exchange rate changes from the first stage regression. The set of instruments Z_t consists of a constant, the dividend-price ratio, the lagged equity return, plus a measure for the lagged equity return variance, exchange rate return variance, and their covariance. The second stage regression is estimated jointly with the zero stage regression by GMM which allows the standard errors of the second stage regression to incorporate not only the uncertainty deriving from the first-stage regression, but also the one from the zero stage regression. The standard errors are based on the Newey-West estimate of the covariance matrix with maximum lag order set equal to $T^{1/2}$. The J-statistic (Hansen, 1982) plus the according p-value are reported for the null hypothesis that the model is well-specified and the moment conditions do hold. The model is estimated for each subsample separately. The shortened full sample (January 1990 to July 2007) consists of 210 observations and the second subsample (January 1999 to July 2007) consists of 103 observations. The standard errors are reported in square brackets. ***, **, and * denote significance levels of 1, 5, and 10%, respectively.

C.5 Market Capitalization Weighted CHF Index

As an extension, this section provides the first and second stage results for a financially weighted CHF index, namely an MSCI market capitalization-weighted exchange rate and interest rate index based on the same weights as the USD indices. Overall, the results are similar to the ones of the trade-weighted CHF exchange rate. The slightly different pattern in the predicted conditional covariance (see Figure A.7) can be explained by the much higher weight that is now attributed to the USD, which is itself considered to be a safe currency.

Concerning the price of risk estimates, the findings are the same as for the other CHF exchange rates (see Table A.11): Proxying the expected exchange rate change by the prediction of the zero stage regression yields more realistic and reliable estimations of the price of risk as compared to measuring the expected exchange rate change by the actual ex-post exchange rate.

Figure A.7: Predicted Conditional Covariance - Market Capitalization Weighted CHF Index

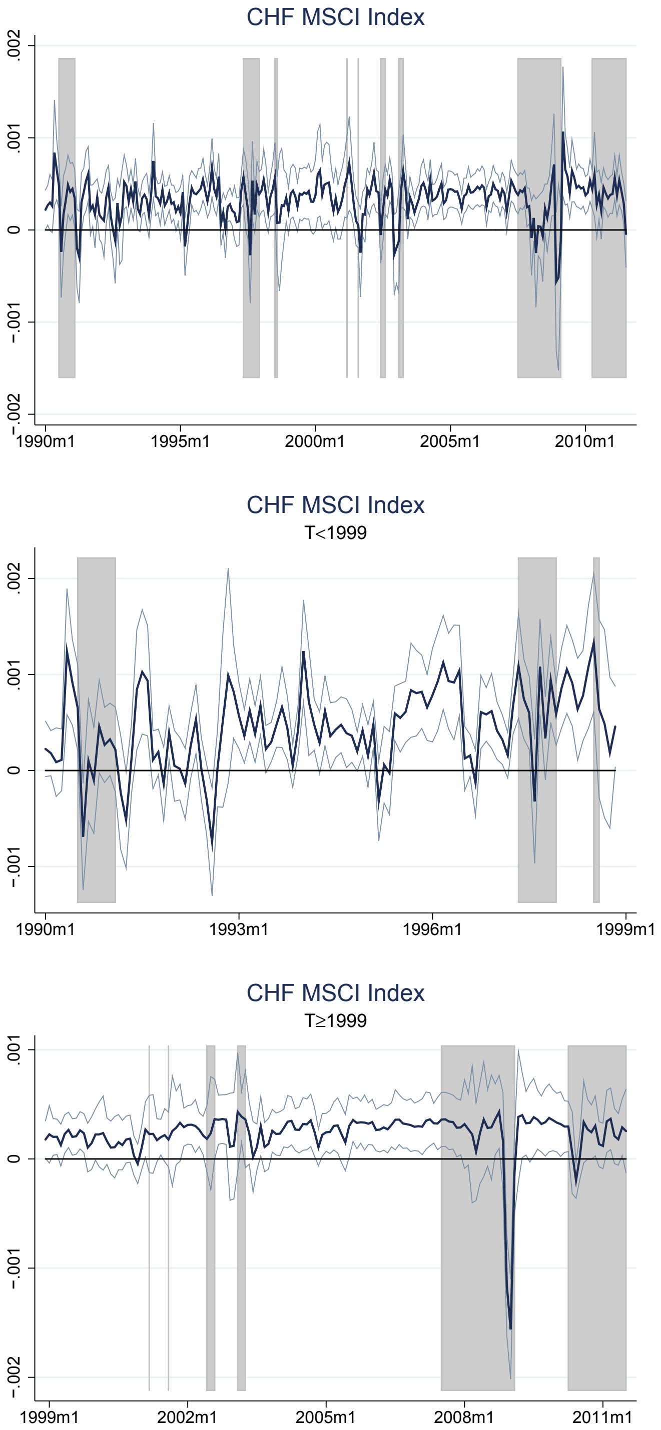

Notes: The estimates of the conditional covariance correspond to the fitted value of the first stage regression: $\widehat{Cov_t}\left(r^{\omega}_{t+1}, \triangle e_{t+1}\right) = \hat{\alpha}_Z Z_t$ (see equation (9)). In this first stage regression, the ex-post covariance obtained from the zero stage regressions is regressed on a set of instruments. The set of instruments Z_t consists of a constant, the dividend-price ratio, the lagged equity return, plus a measure for the lagged equity return variance, exchange rate return variance, and their covariance. The two thin lines represent the 95% confidence band and are based on a two sided t-statistic with Newey-West estimates of the standard errors.

Table A.11: Second Stage Regression - Market Capitalization Weighted CHF Index

$r^*_{f,t+1} + E_t[\triangle e_{t+1}] - r_{f,t+1}$	$E_t[\triangle e_{t+1}] = \triangle e_{t+1}$			$E_t[\triangle e_{t+1}] = 0$			$E_t[\triangle e_{t+1}] = E_t\widehat{[\triangle e_{t+1}]}$		
$+\frac{1}{2}\widetilde{Var_t}(\triangle e_{t+1})$	*all*	$T < 1999$	$T \geq 1999$	*all*	$T < 1999$	$T \geq 1999$	*all*	$T < 1999$	$T \geq 1999$
$\widetilde{Cov_t}(r^{\omega}_{t+1}, \triangle e_{t+1})$	-10.047*	0.809	-11.317**	0.814*	1.247**	0.035	2.127	5.641***	0.085
	[6.773]	[4.599]	[5.236]	[0.505]	[0.553]	[0.203]	[1.679]	[2.260]	[2.179]
$Cons$	0.002	-0.000	0.002	0.001***	0.001**	0.001***	-0.001**	-0.002	-0.001*
	[0.002]	[0.003]	[0.002]	[0.000]	[0.000]	[0.000]	[0.001]	[0.002]	[0.001]
J-statistic	6.892	6.560	2.895	6.232	5.665	4.809	6.256	6.608	3.755
p-value	0.142	0.161	0.575	0.182	0.226	0.307	0.181	0.158	0.440

Notes: This table reports the results of the second stage regression for the case of no time variation in the price of risk (see equation (13)). The dependent variables are the USD and CHF safety premium, respectively, defined as the expected excess return of investing in the foreign risk-free asset by shorting the home risk-free asset. The expected exchange rate change used to calculate this expected excess return is proxied first by the actual exchange rate change, then by zero, and finally by the fitted value of the zero stage regression. The regressors are a constant and the estimate of the conditional covariance between stock returns and exchange rate changes from the first stage regression. The set of instruments Z_t consists of a constant, the dividend-price ratio, the lagged equity return, plus a measure for the lagged equity return variance, exchange rate return variance, and their covariance. The second stage regression is estimated jointly with the zero stage regression by GMM which allows the standard errors of the second stage regression to incorporate not only the uncertainty deriving from the first-stage regression, but also the one from the zero stage regression. The standard errors are based on the Newey-West estimate of the covariance matrix with maximum lag order set equal to $T^{1/2}$. The J-statistic (Hansen, 1982) plus the according p-value are reported for the null hypothesis that the model is well-specified and the moment conditions do hold. The number of observations is 259 for the full sample, 107 for the first and 152 for the second subsample. The standard errors are reported in square brackets. ***, **, and * denote significance levels of 1, 5, and 10%, respectively.

C.6 Sample Beginning in 1975

By complementing the interbank rates data with euro currency deposit rates data (Datastream) and replacing the local stock market indices by the MSCI World index (converted into the respective currency), the sample can be extended to go back to January 1975 (which corresponds to a total of 439 observations). This section reports the according first and second stage results.

Unlike in the benchmark case with the local stock market indices, one has now to be aware of the fact that the covariance between the exchange rate and the global stock market index converted into the respective currency incorporates direct exchange rate effects. For illustration, think of a situation where the local currency appreciates, while the global stock market index remains stable. In that case, the covariance between the exchange rate and the global stock market index converted into the local currency is positive even tough the value of the global stock market index has not changed. In the case of a safe currency, this implies that the covariance will tend to be overestimated. The predicted conditional covariance estimates plotted in Figure A.8 are indeed higher than the ones in the benchmark case reported in the main body of the paper (see Figure 5). The price of risk estimates, on the other hand, are lower than the findings for the two subperiods in the benchmark case (see Table 7). Overall, however, the findings are the same as for the other CHF exchange rates: Proxying the expected exchange rate change by the prediction of the zero stage regression yields at least as or more realistic and reliable estimations of the price of risk as compared to measuring the expected exchange rate change by the actual ex-post exchange rate.

Figure A.8: Predicted Conditional Covariance - Sample Beginning in 1975

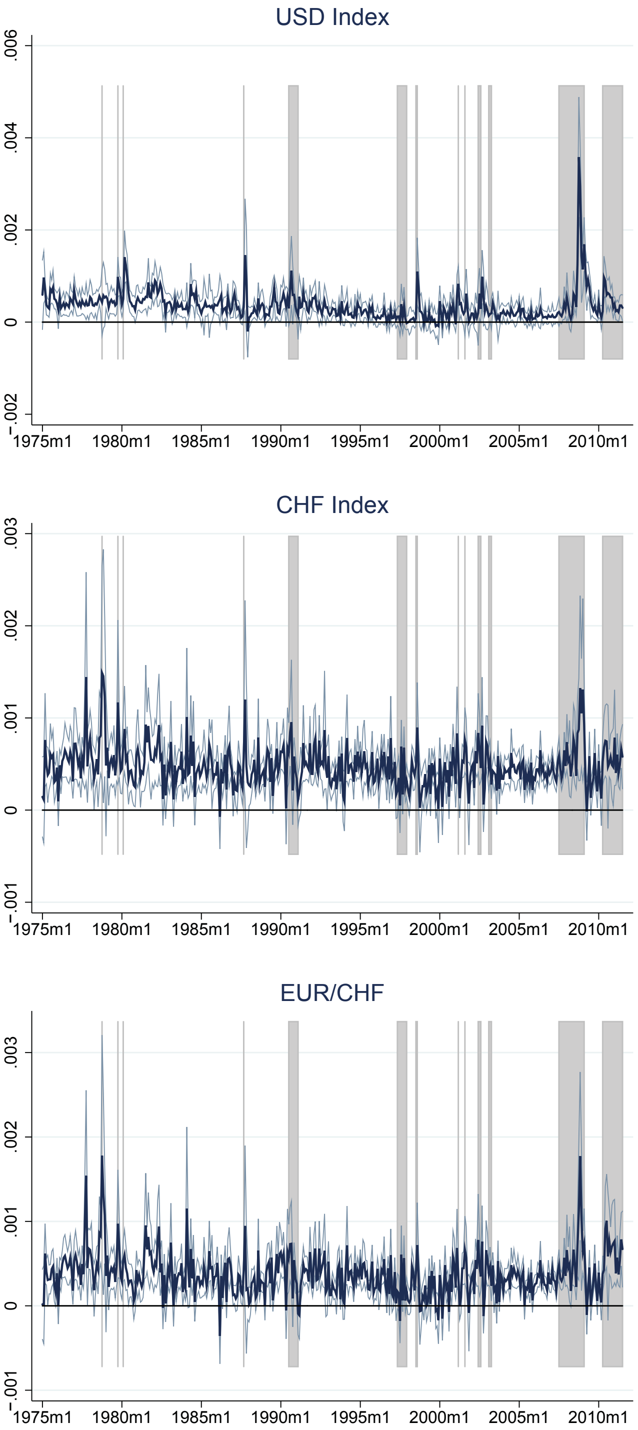

Notes: The estimates of the conditional covariance correspond to the fitted value of the first stage regression: $\widehat{Cov}_t\left(r^{\omega}_{t+1}, \triangle e_{t+1}\right) = \hat{\alpha}_Z Z_t$ (see equation (9)). In this first stage regression, the ex-post covariance obtained from the zero stage regressions is regressed on a set of instruments. The set of instruments Z_t consists of a constant, the dividend-price ratio, the lagged equity return, plus a measure for the lagged equity return variance, exchange rate return variance, and their covariance. The two thin lines represent the 95% confidence band and are based on a two sided t-statistic with Newey-West estimates of the standard errors.

Table A.12: Second Stage Regression - Sample Beginning in 1975

$r^*_{f,t+1} + E_t[\triangle e_{t+1}] - r_{f,t+1}$	$E_t[\triangle e_{t+1}] = \triangle e_{t+1}$			$E_t[\triangle e_{t+1}] = 0$			$E_t[\triangle e_{t+1}] = E_t\widehat{[\triangle e_{t+1}]}$		
$+\frac{1}{2}\widetilde{Var}_t(\triangle e_{t+1})$	USD Index	CHF Index	EUR/CHF	USD Index	CHF Index	EUR/CHF	USD Index	CHF Index	EUR/CHF
$\widetilde{Cov}_t\left(r^{\omega}_{t+1}, \triangle e_{t+1}\right)$	6.086*	-0.830	3.212	0.681**	1.147**	0.636*	0.953	1.151*	0.079
	[4.063]	[5.360]	[2.812]	[0.359]	[0.530]	[0.482]	[0.927]	[0.763]	[0.557]
Cons	-0.001	0.000	-0.001	0.000*	0.001***	0.002***	0.001*	-0.001***	-0.000*
	[0.002]	[0.003]	[0.001]	[0.000]	[0.000]	[0.000]	[0.001]	[0.000]	[0.000]
J-statistic	2.087	3.729	1.520	4.827	8.218	8.802	8.840	8.449	9.714
p-value	0.720	0.444	0.823	0.306	0.084	0.066	0.065	0.076	0.046

Notes: This table reports the results of the second stage regression for the case of no time variation in the price of risk (see equation (13)). The dependent variables are the USD and CHF safety premium, respectively, defined as the expected excess return of investing in the foreign risk-free asset by shorting the home risk-free asset. The expected exchange rate change used to calculate this expected excess return is proxied first by the actual exchange rate change, then by zero, and finally by the fitted value of the zero stage regression. The regressors are a constant and the estimate of the conditional covariance between stock returns and exchange rate changes from the first stage regression. The set of instruments Z_t consists of a constant, the dividend-price ratio, the lagged equity return, plus a measure for the lagged equity return variance, exchange rate return variance, and their covariance. The second stage regression is estimated jointly with the zero stage regression by GMM which allows the standard errors of the second stage regression to incorporate not only the uncertainty deriving from the first-stage regression, but also the one from the zero stage regression. The standard errors are based on the Newey-West estimate of the covariance matrix with maximum lag order set equal to $T^{1/2}$. The J-statistic (Hansen, 1982) plus the according p-value are reported for the null hypothesis that the model is well-specified and the moment conditions do hold. The number of observations is 439 (January 1975 to August 2011). The standard errors are reported in square brackets. ***, **, and * denote significance levels of 1, 5, and 10%, respectively.

C.7 Sample Beginning in 1987

Historical data on the Swiss Performance Index (SPI) goes back to 1987. At the cost of a lower number of countries that can be included in the interest rate index, the beginning of the sample can be shifted from 1990 to 1987 (which corresponds to a total of 280 observations). This section reports the according first and second stage results. They hardly differ from the benchmark results in the main body of the paper.

Figure A.9: Predicted Conditional Covariance - Sample Beginning in 1987

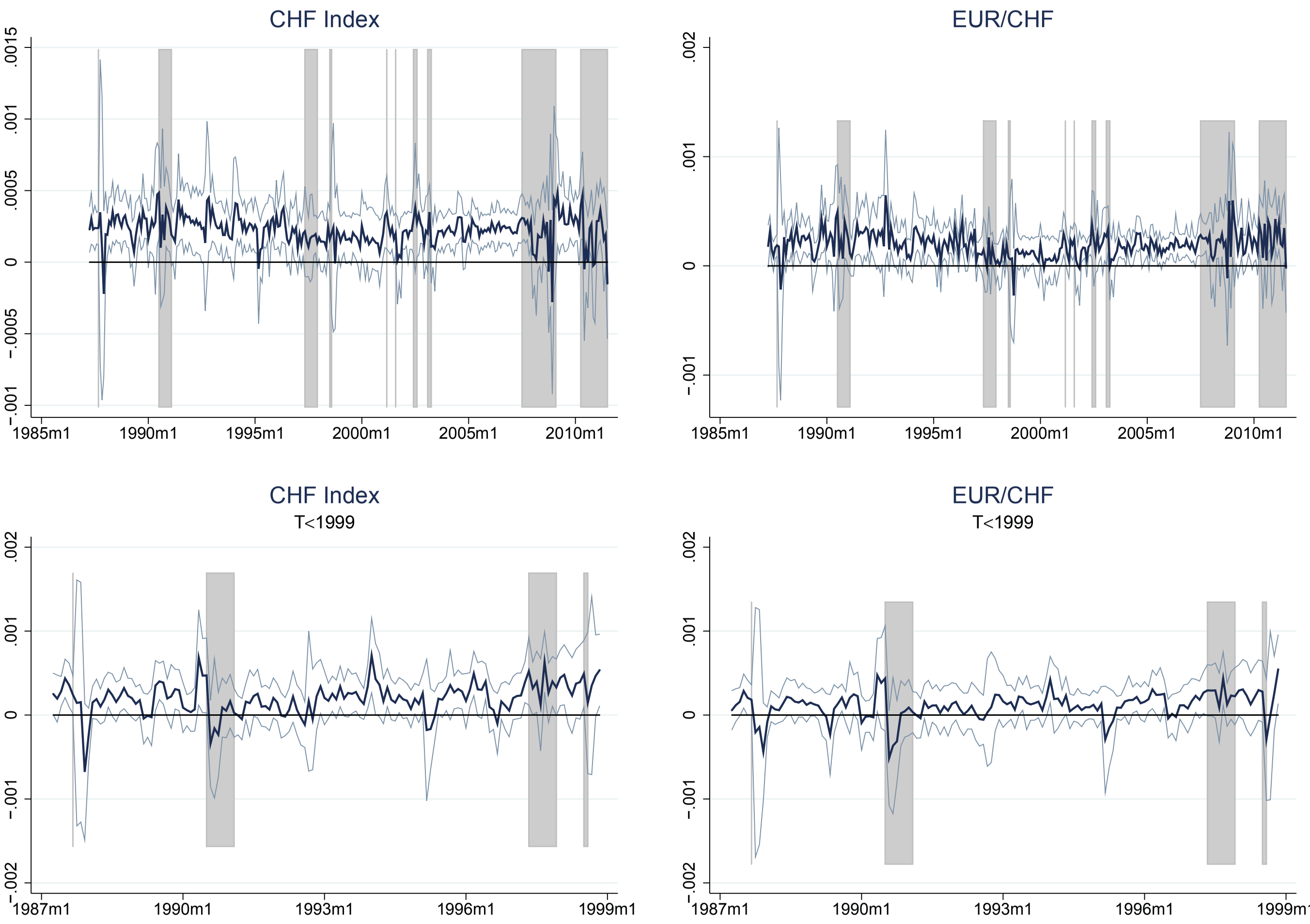

Notes: The estimates of the conditional covariance correspond to the fitted value of the first stage regression: $\widehat{Cov_t}\left(r^{\omega}_{t+1}, \triangle e_{t+1}\right) = \hat{\alpha}_Z Z_t$ (see equation (9)). In this first stage regression, the ex-post covariance obtained from the zero stage regressions is regressed on a set of instruments. The set of instruments Z_t consists of a constant, the dividend-price ratio, the lagged equity return, plus a measure for the lagged equity return variance, exchange rate return variance, and their covariance. The two thin lines represent the 95% confidence band and are based on a two sided t-statistic with Newey-West estimates of the standard errors.

Table A.13: Second Stage Regression - Sample Beginning in 1987

Full Sample

$r^{*}_{f,t+1} + E_t[\triangle e_{t+1}] - r_{f,t+1} + \frac{1}{2}\widetilde{Var}_t(\triangle e_{t+1})$	$E_t[\triangle e_{t+1}] = \triangle e_{t+1}$ CHF Index	$E_t[\triangle e_{t+1}] = \triangle e_{t+1}$ EUR/CHF	$E_t[\triangle e_{t+1}] = 0$ CHF Index	$E_t[\triangle e_{t+1}] = 0$ EUR/CHF	$E_t[\triangle e_{t+1}] = \widehat{E_t[\triangle e_{t+1}]}$ CHF Index	$E_t[\triangle e_{t+1}] = \widehat{E_t[\triangle e_{t+1}]}$ EUR/CHF
$\widetilde{Cov}_t(r^{\omega}_{t+1}, \triangle e_{t+1})$	-22.778***	-13.666**	-1.277**	-0.794*	-7.509**	-0.954
	[9.541]	[7.604]	[0.749]	[0.550]	[4.393]	[2.202]
Cons	0.005**	0.003**	0.002***	0.002***	0.002	0.000
	[0.002]	[0.001]	[0.000]	[0.000]	[0.001]	[0.001]
J-statistic	0.812	1.495	2.045	2.995	1.890	4.487
p-value	0.937	0.827	0.727	0.559	0.756	0.344

Subperiod 1

$r^{*}_{f,t+1} + E_t[\triangle e_{t+1}] - r_{f,t+1} + \frac{1}{2}\widetilde{Var}_t(\triangle e_{t+1})$	$E_t[\triangle e_{t+1}] = \triangle e_{t+1}$ CHF Index	$E_t[\triangle e_{t+1}] = \triangle e_{t+1}$ EUR/CHF	$E_t[\triangle e_{t+1}] = 0$ CHF Index	$E_t[\triangle e_{t+1}] = 0$ EUR/CHF	$E_t[\triangle e_{t+1}] = \widehat{E_t[\triangle e_{t+1}]}$ CHF Index	$E_t[\triangle e_{t+1}] = \widehat{E_t[\triangle e_{t+1}]}$ EUR/CHF
$\widetilde{Cov}_t(r^{\omega}_{t+1}, \triangle e_{t+1})$	-8.493*	-4.758	0.517*	0.822*	3.845**	3.077*
	[5.287]	[5.531]	[0.350]	[0.519]	[2.092]	[1.904]
Cons	0.003*	0.002**	0.002***	0.002***	0.002**	0.001***
	[0.002]	[0.001]	[0.000]	[0.000]	[0.001]	[0.001]
J-statistic	4.149	2.506	3.986	1.967	4.274	1.926
p-value	0.386	0.644	0.408	0.742	0.370	0.749

Notes: This table reports the results of the second stage regression for the case of no time variation in the price of risk (see equation (13)). The dependent variables are the USD and CHF safety premium, respectively, defined as the expected excess return of investing in the foreign risk-free asset by shorting the home risk-free asset. The expected exchange rate change used to calculate this expected excess return is proxied first by the actual exchange rate change, then by zero, and finally by the fitted value of the zero stage regression. The regressors are a constant and the estimate of the conditional covariance between stock returns and exchange rate changes from the first stage regression. The set of instruments Z_t consists of a constant, the dividend-price ratio, the lagged equity return, plus a measure for the lagged equity return variance, exchange rate return variance, and their covariance. The second stage regression is estimated jointly with the zero stage regression by GMM which allows the standard errors of the second stage regression to incorporate not only the uncertainty deriving from the first-stage regression, but also the one from the zero stage regression. The standard errors are based on the Newey-West estimate of the covariance matrix with maximum lag order set equal to $T^{1/2}$. The J-statistic (Hansen, 1982) plus the according p-value are reported for the null hypothesis that the model is well-specified and the moment conditions do hold. The model is estimated for each subsample separately. The extended full sample (May 1987 to August 2011) consists of 280 observations and the second subsample (May 1987 to August 2011) consists of 140 observations. The standard errors are reported in square brackets. ***, **, and * denote significance levels of 1, 5, and 10%, respectively.

C.8 "Replicating" Maggiori (2013)

Maggiori (2013) partly uses data that is not publicly available (for example, the data on market capitalization by MSCI used as weights in the construction of the exchange rate and interest rate indices). Furthermore, he does not specify what interest rate data he is using. For this reason, I am not able to do an exact replication of his results. Here, I present the results obtained when using the same stock market index (MSCI World) and the same time span (April 1975 to March 2010, i.e. 420 observations) by complementing the interbank rates data with euro currency deposit rates data (Datastream). The second stage estimates are the result of iterated GMM. The results are close to the ones reported by Maggiori.

C.8.1 Zero Stage Regressions

Table A.14: Equity Returns

r^{ω}_{t+1}	MSCI World
dp_t	0.009*
	[0.005]
r^{ω}_{t}	0.117
	[0.077]
$Cons$	0.040**
	[0.018]
R^2	0.020

Table A.15: Exchange Rate Returns

$\triangle e_{t+1}$	
$r^{*}_{f,t+1} - r_{f,t+1}$	1.213
	[0.825]
$\triangle e_t$	0.059
	[0.058]
$Cons$	0.000
	[0.001]
R^2	0.012

Figure A.10: Ex-Post Covariance

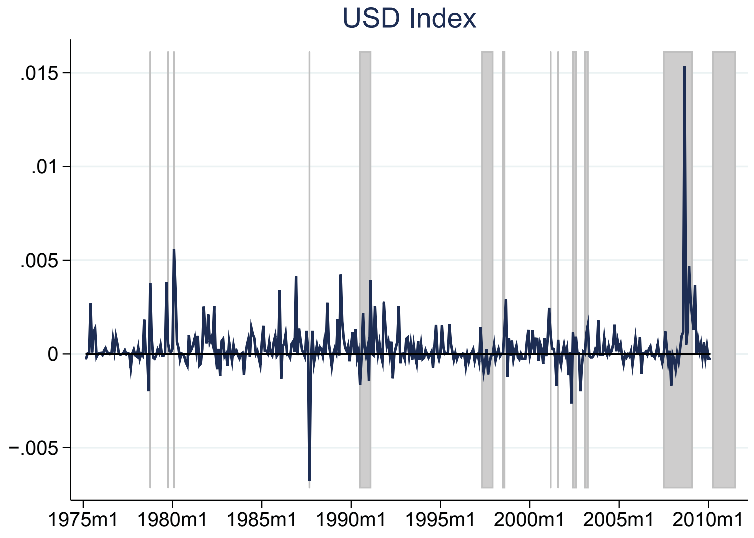

C.8.2 First Stage Regression

Table A.16: First Stage Regression

	$\widetilde{Cov}_t\left(r^{\omega}_{t+1}, \triangle e_{t+1}\right)$
dp_t	0.000*
	[0.000]
r^{ω}_t	-0.002
	[0.002]
$\triangle e_t$	0.001
	[0.003]
var'^{e}_t	-0.003
	[0.040]
var'^{r}_t	0.027**
	[0.013]
cov'_t	0.050*
	[0.027]
$Cons$	0.001***
	[0.000]
R^2	0.066
F-statistic	13.100
χ^2-statistic	78.600
p-value (χ^2-stat.)	0.000

Figure A.11: Conditional Covariance

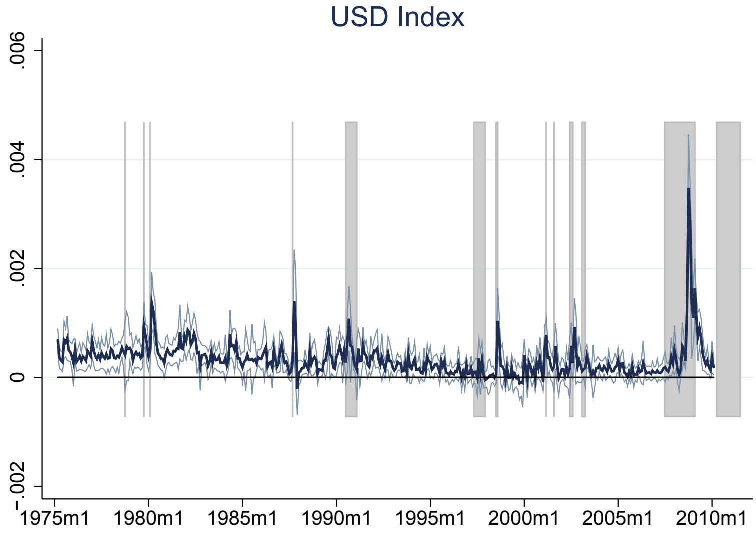

C.8.3 Second Stage Regression

Table A.17: Second Stage Regression

	$r^*_{f,t+1} + \triangle e_{t+1} - r_{f,t+1} + \frac{1}{2}\widetilde{Var}_t\left(\triangle e_{t+1}\right)$
$\widetilde{Cov}_t\left(r^{\omega}_{t+1}, \triangle e_{t+1}\right)$	8.121**
	[4.286]
$Cons$	-0.001
	[0.002]

C.8.4 Episodes of Crisis - Extended

Table A.18: Episodes of Crisis (Stock Market Volatility Shocks) - Extended

Event	Period
OPEC II, Fed Currency Intervention	November 1978
Iran Hostage Crisis	November 1979
Silver Wednesday, US-Iran Military Intervention	March 1980
1987 Crash - Black Monday	October 1987
Gulf War I	August 1990 - March 1991
Asian Crisis	June 1997 - January 1998
Russian Crisis, LTCM Default	August - September 1998
Dotcom Bust*	April 2001
9/11 Terrorist Attacks	September 2001
Worldcom, Enron Bankruptcy	July - September 2002
Gulf War II	March - May 2003
Credit Crunch, Lehman Default	August 2007 - March 2009, October 2008
Greek Government-Debt Crisis*	May 2010 - end of sample

All episodes except the ones marked by * are taken from Bloom (2009). Bloom identifies periods of major stock market volatility shocks by analysing the deviations of a stock market volatility series from its detrended mean. I partially extend the length of these periods as the events leading to this increased volatility in stock markets already started earlier and lasted longer than indicated by Bloom and because there is evidence in my time series of extensive market reaction. I followed Maggiori (2013) in adding the event of the dotcom bust, and finally included the recent Greek government-debt crisis.

D GARCH - Technical Details and Complete Results

Early specifications of multivariate GARCH (MGARCH) models like the so-called VEC model by Bollerslev et al. (1988) are very general and not convenient when to be put into practice, amongst other things due to the large number of parameters that needs to be estimated. Various, more parsimonious MGARCH models have been proposed to make parameter estimation more feasible while still allowing to capture complex processes for the conditional covariance. A popular specification is the Dynamic Conditional Correlation (DCC) model by Engle (2002). It is based upon the decomposition of the conditional covariance matrix into conditional standard deviations and correlations that are then modelled separately. It is this specification that I employ.

D.1 GARCH - constant price of risk

D.1.1 Safety Premium in a MGARCH framework

To see how the theoretical safety premium model above can be estimated by a multivariate GARCH model, note that under complete markets, it should not make a difference whether a home investor shorting the home risk free asset goes long on the foreign risk free asset or on the home market portfolio. In both cases, the expected discounted return should be zero:

$$\begin{cases} 0 &= E_t\left[M_{t+1}\left(R^*_{t+1}\frac{\mathcal{E}_{t+1}}{\mathcal{E}_t} - R_{t+1}\right)\right] \\ 0 &= E_t\left[M_{t+1}\left(R^{\omega}_{t+1} - R_{t+1}\right)\right]. \end{cases} \tag{A.1}$$

Linearizing the system of equations (A.1) suggests the following expressions for expected excess returns:

$$\begin{cases} E_t\left[r^*_{f,t+1} + \triangle e_{t+1} - r_{f,t+1}\right] &= b_t Cov_t\left(r^{\omega}_{t+1} - r_{f,t+1}, r^*_{f,t+1} + \triangle e_{t+1} - r_{f,t+1}\right) \\ E_t\left[r^{\omega}_{t+1} - r_{f,t+1}\right] &= b_t Var_t\left(r^{\omega}_{t+1} - r_{f,t+1}\right). \end{cases} \tag{A.2}$$

The first equation in (A.2) corresponds to the already familiar expression for the currency safety premium. The second equation says that the risk premium that the market portfolio return has to offer is equal to the price of risk multiplied by its own conditional variance.

This system of equations can be written in matrix form with xr_{t+1} standing for the vector of excess returns:

$$xr_{t+1} = \mu_{t+1} + u_{t+1}, \tag{A.3}$$

where $\mu_{t+1} = Cx_{t+1}$ and the u_{t+1}s are an exogenous martingale difference sequence with conditional

variance covariance matrix H_{t+1}:

$$xr_{t+1} = \begin{bmatrix} xr^c_{t+1} \\ xr^m_{t+1} \end{bmatrix} = \begin{bmatrix} r^*_{f,t+1} + \triangle e_{t+1} - r_{f,t+1} \\ r^\omega_{t+1} - r_{f,t+1} \end{bmatrix}, \quad H_{t+1} = \begin{bmatrix} h_1 & h_2 \end{bmatrix} = \begin{bmatrix} \sigma^2_{c,t+1} & \sigma_{c,m,t+1} \\ \sigma_{c,m,t+1} & \sigma^2_{m,t+1} \end{bmatrix} \quad \text{(A.4)}$$

$$x_{t+1} = \begin{bmatrix} 1 \\ h_2 \end{bmatrix}, \quad C = \begin{bmatrix} \gamma^c_0 & \gamma^c_1 & 0 \\ \gamma^m_0 & 0 & \gamma^m_1 \end{bmatrix} \quad \Rightarrow \quad \mu_{t+1} = \begin{bmatrix} \mu^c_{t+1} \\ \mu^m_{t+1} \end{bmatrix} = \begin{bmatrix} \gamma^c_0 + \gamma^c_1 \sigma_{c,m,t+1} \\ \gamma^m_0 + \gamma^m_1 \sigma^2_{m,t+1} \end{bmatrix} \quad \text{(A.5)}$$

μ^c_{t+1} is equal to the expected excess return of investing abroad and thus the currency safety premium. The coefficient that corresponds to the price of risk b_t and thus the coefficient of interest here is γ^c_1.[23] I will first treat it as constant as I did in the GMM analysis, and later allow it to be time-varying.[24]

In the Dynamic Conditional Correlation (DCC) model by Engle (2002), u_{t+1} is assumed to follow a normal distribution. The conditional covariance matrix H_{t+1} is decomposed into a matrix of conditional variances D_{t+1} and a matrix of conditional (quasi-)correlations R_{t+1}:[25]

$$H_{t+1} = D^{1/2}_{t+1} R_{t+1} D^{1/2}_{t+1} \quad \text{(A.6)}$$

The conditional variances are modelled as univariate GARCH(1,1) processes:

$$D_{t+1} = \begin{bmatrix} \sigma^2_{c,t+1} & 0 \\ 0 & \sigma^2_{m,t+1} \end{bmatrix}, \quad \text{where} \quad \sigma^2_{i,t+1} = s_i + \alpha_i u^2_{i,t} + \beta \sigma^2_{i,t}. \quad \text{(A.7)}$$

The conditional covariances are modelled as a nonlinear function of these conditional variances. The conditional quasicorrelation parameters that weight the nonlinear combination of the conditional variances also follow a GARCH-like process:

$$R_{t+1} = diag(Q_{t+1})^{-1/2} Q_{t+1} diag(Q_{t+1})^{-1/2} \quad \text{(A.8)}$$

$$Q_{t+1} = (1 - \lambda_1 - \lambda_2) R + \lambda_1 \widetilde{u}_t \widetilde{u}'_t + \lambda_2 Q_t \quad \text{(A.9)}$$

with

$$R_{t+1} = \begin{bmatrix} 1 & \rho_{c,m,t+1} \\ \rho_{c,m,t+1} & 1 \end{bmatrix} \quad \text{(A.10)}$$

being a matrix of conditional quasicorrelations. $\widetilde{u}_{t+1}$ is a vector of standardized residuals: $D^{-1/2}_{t+1} u_{t+1}$.

Given the strong indication of asymmetry in the correlation between changes in the Swiss franc exchange rate and stock market changes and the implied asymmetry in the conditional covariance, I have these models estimated by quasi-maximum likelihood (QML). There is still an active literature developing GARCH models further and suggesting specifications that model in one way or another such asymmetries specifically (see for example Bekaert et al. (2015)). Already the quasi-maximum likelihood estimator, however, allows to estimate MGARCH models consistently without having to worry about about modeling the non-Gaussianity in the shocks (see Fiorentini and Sentana (2007)).

[23] I allow for the currency price of risk γ^c_1 and the market price of risk γ^m_1 to be different as these two prices might reflect some different other factors that are not controlled for.

[24] Even tough equation (A.2) does not contain constants, I include them in order to be consistent with the GMM estimation.

[25] As stated by Engle (2009) and Aielli (2011), the parameters in R_{t+1} are not standardized to be correlations and are thus known as quasicorrelations.

D.1.2 MGARCH results

During the estimation process for the first subsample, I encountered some convergence problems, which are a common issue of GARCH models when put into practice (see for example Silvennoinen and Teräsvirta (2009)), so I only present results for the full sample and the second subsample. Let us first have a look at the conditional covariances implied by these GARCH models which are pictured in Figure A.12. In the case of the USD, its evolution is pretty comparable to the one estimated with instruments. In the case of the CHF, across all exchange rates and samples it now looks much closer to what I would expect, with clear peaks in crisis episodes. Altogether, the estimates for the conditional covariance implied by the GARCH models seem to be more convincing than the ones calculated with instruments.

The second object of interest are the price of risk estimates (see the first row of Table A.19). They are all positive, even though insignificant. Overall, they are roughly comparable in magnitude to my three-step GMM estimates when using the zero-stage prediction to measure the expected exchange rate change (recall the values of the last and second to last column in Table 7) and thus support this solution to the measurement error problem. Based on the second period GARCH estimates, the safety premium for the Swiss franc reflected in the EUR/CHF exchange rate would be 2.5% (on an annual basis) on average and reach its maximum of around 12.5% during the recent financial crisis, thus values that are larger than the ones suggested by my GMM results from section 7.3.

Altogether, however, also this GARCH model finds only weak evidence that investors are rewarded for their exposure to currency risk, which is consistent with earlier GARCH literature. De Santis and Gérard (1998) estimate a BEKK GARCH model to find the magnitude of the premium for currency risk based on the international CAPM and only obtain insignificant results when estimating constant prices of risk.[26]

D.2 GARCH - time-varying price of risk

The time-varying price of risk in the form of $\gamma_{1,t}^c$ and $\gamma_{1,t}^m$ is modelled using a linear function:

$$\gamma_{1,t}^c = \kappa_c Y_t, \quad \gamma_{1,t}^m = \kappa_m Y_t, \tag{A.11}$$

$$Y_t = [1, dp_t, d.ys_t, d.r_{f,t}, baa\_aaa_t], \tag{A.12}$$

where the κs are 1x5 vectors. Y_t corresponds to a set of instruments including a constant, the market index dividend price ratio dp_t and the change in the gap between long-term and short-term interest rates (yield spread) ys_t measured by the yield of 10-year government bond in excess of the 1-month interbank rate. Furthermore, it includes the change in the home risk-free interest

[26]While the GARCH specifications BEKK by Engle and Kroner (1995) and DCC are shown to produce very similar results (see Caporin and McAleer (2008, 2012)), the DCC model is computationally more attractive.

Figure A.12: GARCH Results - Conditional Covariance Estimates

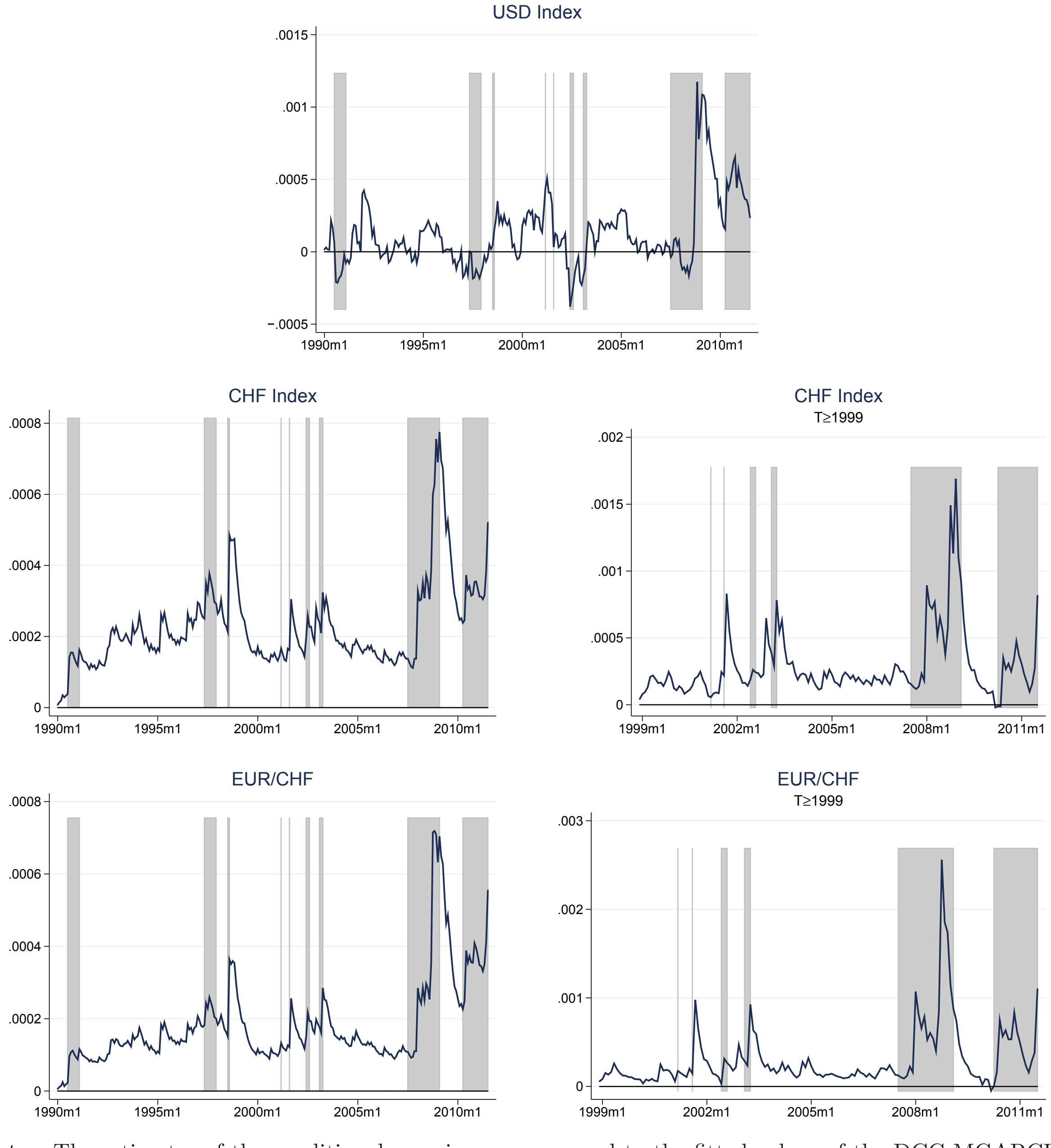

Notes: The estimates of the conditional covariance correspond to the fitted values of the DCC MGARCH-in-mean model for the case of no time variation in the price of risk. For details see section D.1.1.

rate $r_{f,t}$ and the yield difference of Moody's BAA-rated corporate bonds over Moody's AAA-rated corporate bonds baa_aaa_t (taken from FRED), which is used as a measure for default risk. This way of parametrizing the risk price allows to easily check for the time-variation of the coefficient by setting all κ's except the first one (the one related to the constant) equal to zero.

Due to some convergence problems, I was forced to run the model for the USD exchange rate index with only two out of the four instruments. The results in the first six rows of Table A.20 are somewhat more encouraging than the ones from the three-step GMM procedure. For both the USD exchange rate index as well as for the EUR/CHF exchange rate in the second subsample, there are statistically significant coefficients on some of the interaction terms indicating that the price of risk indeed has a time-varying component. Figure A.13 plots the predicted prices of currency risk and the corresponding safety premiums for these two exchange rates. The risk price coefficients see long periods of negative values, especially in the years between 1998 and 2002, a period with multiple major crisis and therefore hardly a time when investors on average were willing to pay extra for risk instead of asking for a compensation. So altogether, this price of risk picture should probably be looked at with caution. The same holds for the safety premium figure for the USD, while the one for the CHF provides convincing values. Based on these estimates, the safety premium for the Swiss franc reflected in the EUR/CHF exchange rate would be 3% on average (on an annual basis) and around 6% up to 46% during the recent financial crisis. These values are considerably larger than what the GMM results from section 7.3 suggest.

But is it really the case that the time-varying price of risk models should be preferred to the constant price of risk models? The better performance of the models with a time-varying risk price goes with a higher complexity of these models, measured by the number of parameters. Trading off fit against complexity in the GARCH models, both the Akaike and Bayesian information criterion suggest going with the constant price of risk version. Thus, restricting the price of risk to be constant seems still to be justified.

Figure A.13: GARCH Results - Time-Varying Price of Risk

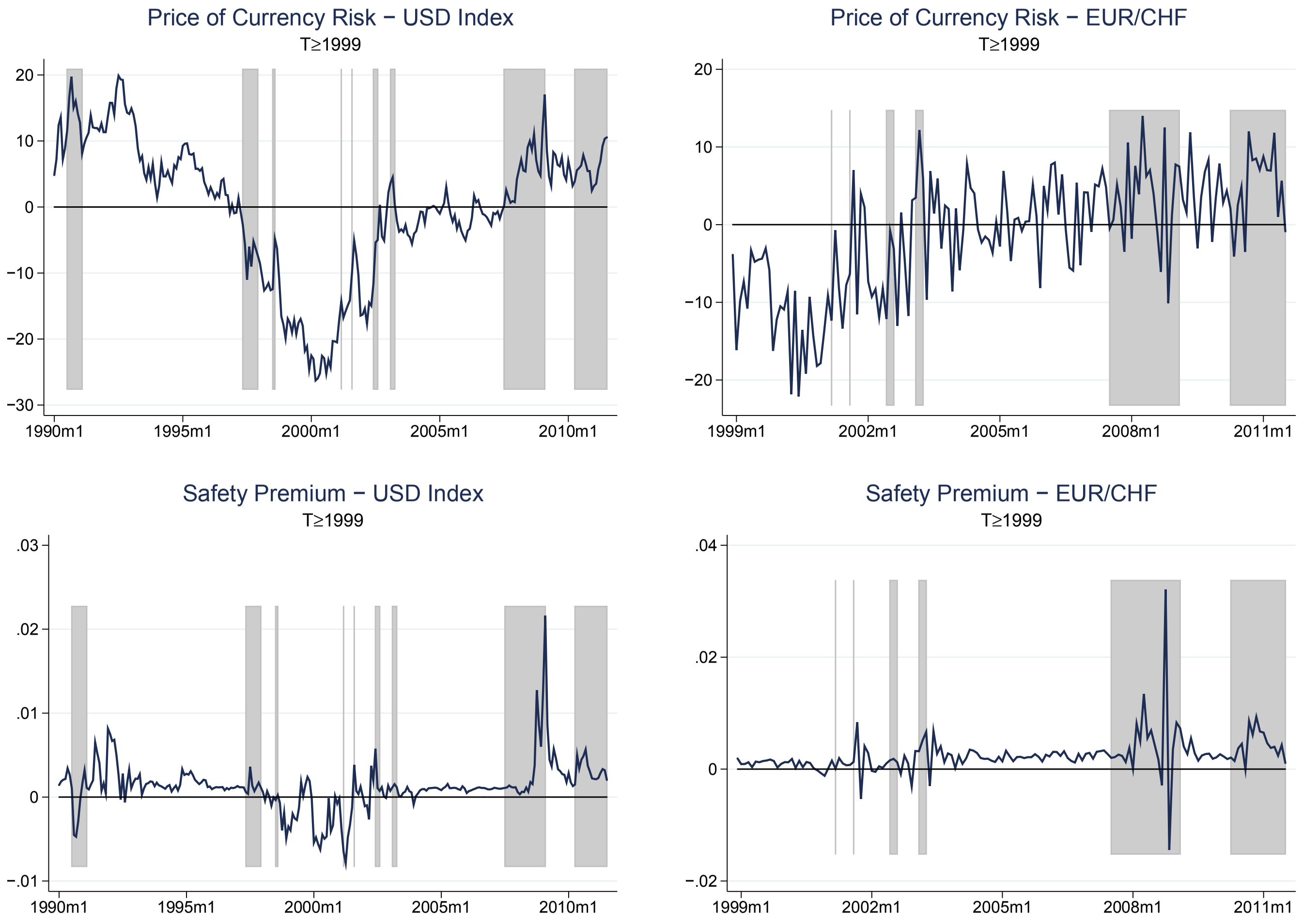

Notes: The estimates of the price of currency risk and the according safety premium (which is equal to the expected excess return of investing in the foreign risk-free bond by shorting the home risk-free bond) correspond to the fitted values of the DCC GARCH model allowing for time variation in the price of risk. For details see section D.1.1. The estimates are based on monthly returns from January 1990 to August 2011. The total number of observations is 259 for the full sample and 152 for the second subsample.

Table A.19: GARCH - Constant Price of Risk

	USD Index	CHF Index		EUR/CHF	
VARIABLES	*all*	*all*	$T \geq 1999$	*all*	$T \geq 1999$
$\boldsymbol{xr^c_{t+1}}$					
$\sigma_{c,m,t+1}$	6.150	3.758	10.168	2.208	3.482
	[6.038]	[10.560]	[6.896]	[11.767]	[3.192]
$Cons$	0.000	-0.000	-0.002	0.001	0.001
	[0.001]	[0.002]	[0.002]	[0.002]	[0.001]
**GARCH process for $\boldsymbol{\sigma^2_{c,t+1}}$**					
$u^2_{c,t}$	-0.052**	0.111***	0.408**	0.121*	0.552**
	[0.024]	[0.043]	[0.161]	[0.068]	[0.226]
$\sigma^2_{c,t}$	-0.384	0.896***	0.542***	0.893***	0.568***
	[0.234]	[0.050]	[0.143]	[0.065]	[0.101]
$Cons$	0.001***	0.000	0.000*	0.000	0.000
	[0.000]	[0.000]	[0.000]	[0.000]	[0.000]
$\boldsymbol{xr^m_{t+1}}$					
$\sigma^2_{m,t+1}$	0.489	-3.310	-1.368	-3.196	-3.034
	[1.719]	[3.021]	[4.035]	[3.069]	[3.870]
$Cons$	0.005	0.012**	0.005	0.012**	0.009
	[0.003]	[0.006]	[0.007]	[0.006]	[0.007]
**GARCH process for $\boldsymbol{\sigma^2_{m,t+1}}$**					
$u^2_{m,t}$	0.157***	0.163**	0.182*	0.157***	0.156***
	[0.055]	[0.064]	[0.096]	[0.054]	[0.052]
$\sigma^2_{m,t}$	0.818***	0.739***	0.714***	0.748***	0.761***
	[0.049]	[0.151]	[0.128]	[0.117]	[0.062]
$Cons$	0.000*	0.000	0.000*	0.000	0.000**
	[0.000]	[0.000]	[0.000]	[0.000]	[0.000]
**Process for (Quasi-)Correlation**					
$Cons$	0.131	0.484***	0.530***	0.498***	0.517***
	[0.136]	[0.089]	[0.119]	[0.091]	[0.117]
λ_1	0.100**	0.008	0.105**	0.006	0.118*
	[0.046]	[0.019]	[0.043]	[0.007]	[0.071]
λ_2	0.811***	0.960***	0.754***	0.965***	0.710***
	[0.091]	[0.023]	[0.078]	[0.009]	[0.099]
Likelihood Function	1100	1180	726.7	1193	737.2
Robust Wald test	1.052	1.448	2.965	1.151	2.179
p-value	0.591	0.485	0.227	0.562	0.336
AIC	-2175	-2334	-1427	-2361	-1448
BIC	-2128	-2288	-1388	-2315	-1409

Notes: This table reports the quasi-maximum likelihood estimates of the DCC GARCH model for the case of no time variation in the price of risk. The mean equation relates the excess return xr_{t+1} of an asset to its risk. xr^c_{t+1} corresponds to the excess return of investing abroad: $xr^c_{t+1} = \gamma^c_0 + \gamma^c_1 \sigma_{c,m,t+1} + u^c_{t+1}$, where γ^c_1 corresponds to the price of currency risk. xr^m_{t+1} is the excess return of investing in the home equity portfolio: $\gamma^m_0 + \gamma^m_1 \sigma^2_{m,t+1} + u^m_{t+1}$, where γ^m_1 corresponds to the price of market risk. The vector of shocks u_{t+1} is $\sim N(0, H_{t+1})$, where H_{t+1} is modelled in the style of DCC and decomposed into a matrix of conditional variances and a matrix or conditional (quasi-)correlations. For details see section D.1.1. The estimates are based on monthly returns from January 1990 to August 2011. The number of observations is 259 for the full sample and 152 for the second subsample. The robust Wald test is reported for the null hypothesis that the price of risk coefficients γ^c_1 and γ^m_1 are jointly equal to zero. Robust standard errors are reported in square brackets. ***, **, and * denote significance levels of 1, 5, and 10%, respectively.

Table A.20: GARCH - Time-Varying Price of Risk

	USD Index	CHF Index		EUR/CHF	
VARIABLES	*all*	*all*	$T \geq 1999$	*all*	$T \geq 1999$
$\boldsymbol{xr^c_{t+1}}$					
$\sigma_{c,m,t+1}$	213.805*	-10.583	17.779	147.409	146.422**
	[114.283]	[88.076]	[114.746]	[115.443]	[67.585]
$\sigma_{c,m,t+1} * dp_t$	53.348*	-3.248	-1.519	35.018	35.490**
	[27.276]	[20.152]	[30.395]	[28.776]	[16.981]
$\sigma_{c,m,t+1} * d.ys_t$		-1,075.587	3,722.933	2,839.150	3,274.112
		[2,621.013]	[3,061.654]	[2,614.545]	[2,207.283]
$\sigma_{c,m,t+1} * d.r_{f,t}$		-2,375.842	4,160.398	992.874	2,477.460
		[2,980.440]	[2,777.950]	[2,318.318]	[1,762.352]
$\sigma_{c,m,t+1} * baa\_aaa_t$	-1,040.716	-177.160	-370.567	-1,177.656	-927.900*
	[1,007.669]	[1,025.611]	[793.450]	[860.192]	[542.029]
Cons	0.001	0.000	-0.004	0.002	0.002
	[0.002]	[0.002]	[0.004]	[0.002]	[0.002]
**GARCH process for $\boldsymbol{\sigma^2_{c,t+1}}$**					
$u^2_{c,t}$	0.022	0.113**	0.438**	0.363	0.525**
	[0.023]	[0.047]	[0.207]	[0.309]	[0.208]
$\sigma^2_{c,t}$	0.887***	0.894***	0.486**	0.668***	0.574***
	[0.079]	[0.053]	[0.198]	[0.178]	[0.106]
Cons	0.000	0.000	0.000*	0.000	0.000
	[0.000]	[0.000]	[0.000]	[0.000]	[0.000]
$\boldsymbol{xr^m_{t+1}}$					
$\sigma^2_{m,t+1}$	64.690**	18.362	59.635	20.105	36.087
	[28.050]	[28.911]	[51.625]	[26.223]	[57.938]
$\sigma^2_{m,t+1} * dp_t$	14.560**	4.946	13.367	5.270	8.820
	[6.911]	[6.709]	[12.252]	[6.215]	[13.901]
$\sigma^2_{m,t+1} * d.ys_t$		-1,148.027	1,194.999	-741.096	1,220.801
		[968.590]	[1,137.763]	[805.792]	[996.426]
$\sigma^2_{m,t+1} * d.r_{f,t}$		-1,029.444	2,063.720*	-691.676	1,873.404
		[1,360.055]	[1,140.929]	[1,014.494]	[1,190.773]
$\sigma^2_{m,t+1} * baa\_aaa_t$	-582.354**	-210.729	-512.876	-212.760	-292.245
	[238.911]	[438.543]	[550.435]	[395.252]	[590.447]
Cons	0.000	0.010*	-0.001	0.010*	0.006
	[0.004]	[0.006]	[0.009]	[0.006]	[0.009]
**GARCH process for $\boldsymbol{\sigma^2_{m,t+1}}$**					
$u^2_{m,t}$	0.143***	0.196	0.162**	0.191**	0.142***
	[0.052]	[0.172]	[0.069]	[0.094]	[0.049]
$\sigma^2_{m,t}$	0.832***	0.680*	0.753***	0.717***	0.786***
	[0.044]	[0.408]	[0.074]	[0.180]	[0.044]
Cons	0.000*	0.000	0.000**	0.000	0.000**
	[0.000]	[0.001]	[0.000]	[0.000]	[0.000]
**Process for (Quasi-)Correlation**					
Cons	0.095	0.500***	0.517***	0.517***	0.538***
	[0.092]	[0.083]	[0.118]	[0.187]	[0.130]
λ_1	0.171*	0.008	0.096*	0.044	0.119*
	[0.091]	[0.014]	[0.049]	[0.057]	[0.070]
λ_2	0.478**	0.961***	0.758***	0.922***	0.744***
	[0.206]	[0.017]	[0.121]	[0.096]	[0.116]
Likelihood Function	1102	1183	729.9	1197	740.9
Robust Wald test	15.49	7.928	10.86	12.73	20.04
p-value	0.017	0.636	0.369	0.239	0.029
AIC	-2175	-2327	-1422	-2355	-1444
BIC	-2121	-2260	-1364	-2288	-1386

Notes: This table reports the quasi-maximum likelihood estimates of the DCC GARCH model allowing for time variation in the price of risk. The mean equation relates the excess return xr_{t+1} of an asset to its risk. xr^c_{t+1} corresponds to the excess return of investing abroad: $xr^c_{t+1} = \gamma^c_0 + \gamma^c_{1,t}\sigma_{c,m,t+1} + u^c_{t+1}$, where $\gamma^c_{1,t} = \kappa_c Y_t$ corresponds to the price of currency risk. xr^m_{t+1} is the excess return of investing in the home equity portfolio: $\gamma^m_0 + \gamma^m_{1,t}\sigma^2_{m,t+1} + u^m_{t+1}$, where $\gamma^m_{1,t} = \kappa_m Y_t$ corresponds to the price of market risk. The vector of instruments Y_t contains a constant, the dividend price ratio, the yield spread between long- and short-term interest rates, the change in the home risk-free interest rate and the yield difference between BAA- and AAA-rated bonds. The vector of shocks u_{t+1} is $\sim N(0, H_{t+1})$, where H_{t+1} is modelled in the style of DCC and decomposed into a matrix of conditional variances and a matrix or conditional (quasi-)correlations. For details see section D.1.1. The estimates are based on monthly returns from January 1990 to August 2011. The number of observations is 259 for the full sample and 152 for the second subsample. The robust Wald test is reported for the null hypothesis that the price of risk coefficients $\gamma^c_{1,t}$ and $\gamma^m_{1,t}$ are jointly equal to zero. Robust standard errors are reported in square brackets. ***, **, and * denote significance levels of 1, 5, and 10%, respectively.
